# Cell shape dynamics during the staphylococcal cell cycle

**DOI:** 10.1038/ncomms9055

**Published:** 2015-08-17

**Authors:** João M. Monteiro, Pedro B. Fernandes, Filipa Vaz, Ana R. Pereira, Andreia C. Tavares, Maria T. Ferreira, Pedro M. Pereira, Helena Veiga, Erkin Kuru, Michael S. VanNieuwenhze, Yves V. Brun, Sérgio R. Filipe, Mariana G. Pinho

**Affiliations:** 1 Laboratory of Bacterial Cell Biology, Instituto de Tecnologia Química e Biológica António Xavier, Universidade Nova de Lisboa, 2780-157 Oeiras, Portugal; 2 Laboratory of Bacterial Cell Surfaces and Pathogenesis, Instituto de Tecnologia Química e Biológica António Xavier, Universidade Nova de Lisboa, 2780-157 Oeiras, Portugal; 3 Department of Chemistry, Indiana University Bloomington, Bloomington, Indiana 47405, USA; 4 Department of Biology, Indiana University Bloomington, Bloomington, Indiana 47405, USA

## Abstract

*Staphylococcus aureus* is an aggressive pathogen and a model organism to study cell division in sequential orthogonal planes in spherical bacteria. However, the small size of staphylococcal cells has impaired analysis of changes in morphology during the cell cycle. Here we use super-resolution microscopy and determine that *S. aureus* cells are not spherical throughout the cell cycle, but elongate during specific time windows, through peptidoglycan synthesis and remodelling. Both peptidoglycan hydrolysis and turgor pressure are required during division for reshaping the flat division septum into a curved surface. In this process, the septum generates less than one hemisphere of each daughter cell, a trait we show is common to other cocci. Therefore, cell surface scars of previous divisions do not divide the cells in quadrants, generating asymmetry in the daughter cells. Our results introduce a need to reassess the models for division plane selection in cocci.

## Abstract (web summary)

Staphylococci are spherical bacteria that divide in sequential orthogonal planes. Here, the authors use super-resolution microscopy to show that staphylococcal cells elongate before dividing, and that the division septum generates less than one hemisphere of each daughter cell, generating asymmetry.

## Graphical abstract

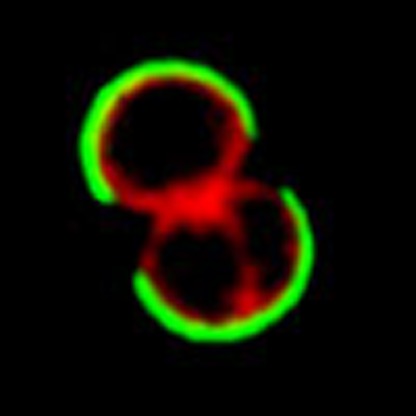

S*taphylococci* are spherical organisms that divide sequentially in three orthogonal planes over three consecutive division cycles^1^,^2^. This mode of division is less common in bacterial cells than equatorial division, observed in many genera. Division in three planes implies that cells retain information about the positioning of the two preceding divisions to divide with precision. Given that this spatial information varies with each division, it cannot be encoded by DNA^3^. Peptidoglycan, the major component of the bacterial cell wall, has been proposed to encode epigenetic information in the form of protuberant, ring-like structures that mark previous division planes and are used by *Staphylococcus aureus* to divide accurately in sequential perpendicular planes^3^.

Orientation of division planes is merely one of the distinctive features of the staphylococcal cell cycle. *S. aureus* has been proposed to have only one cell wall synthesis machine, which incorporates peptidoglycan mostly at the division septum^4^,^5^, while rod-shaped bacteria such as *Escherichia coli* or *Bacillus subtilis* have two major cell wall synthesis machines, one for incorporation of new peptidoglycan at the division septum and another for elongation of the lateral wall^4^. Accordingly, *S. aureus* has only four native Penicillin-Binding Proteins (PBPs 1–4), described to localize at the septum, while *E. coli* and *B. subtilis* have 12 and 16 PBPs, respectively, which localize at the septum or at the lateral wall^4^. PBPs are enzymes involved in the last steps of peptidoglycan biosynthesis, which catalyse the polymerization of the glycan strands, as well as their crosslinking via peptide stems. Given that an elongation-specific machinery seems to be absent in *S. aureus*, increase of the cell surface area required for growth has been attributed to a process of reshaping the flat septum into curved hemispheres of the two daughter cells, which occurs immediately after splitting of the mother cell during division^2^. Reshaping of the flat septum, resulting in doubling of the external surface area of peptidoglycan, could theoretically occur in the absence of synthesis, if accompanied by changes in the angle of the glycan chains with respect to the peptide chains^6^. Alternatively, increase of the surface area could be driven by hydrolysis of peptidoglycan bonds catalysed by specific autolysins. At least 13 genes of the *S. aureus* genome encode known or putative peptidoglycan hydrolases, although the products of only three of these genes (*atl*, *sle1* and *lytM*) have been characterized. Of these genes, *atl* encodes the major autolysin in *S. aureus*, which is involved in the separation of the daughter cells after division^7^,^8^. Interestingly, orthogonal rings of Atl can be observed by immunoelectron microscopy at the surface of *S. aureus* cells, similar to scars of previous divisions^9^, confirming that information regarding the localization of previous, orthogonal, division planes can be present at the cell surface.

*S. aureus* is an aggressive pathogen and one of the most important nosocomial bacteria causing antibiotic-resistant infections. Despite its clinical relevance, the small size of staphylococcal cells (with a ∼1 μm diameter, only four times larger than the diffraction-limited resolution of conventional light microscopy) has impaired a detailed analysis of its cell cycle and of the morphological changes that occur as *S. aureus* grows and divides. This lack of knowledge extends to the cell cycle of other cocci as well. Therefore detailed characterization of the mode of growth and division of *S. aureus* has implications for the global understanding of the cell cycle of cocci. Here, we have used super-resolution microscopy to analyse the dynamics of cell shape and size during the cell cycle of *S. aureus*. We found that, contrary to current thinking, *S. aureus* cells elongate before dividing. Furthermore, we show that the division septum generates less than one hemisphere of each daughter cell and therefore scars of previous divisions do not mark quadrants of the cell. Our results suggest that the models for division plane selection in cocci should be re-examined.

## Results

### *S. aureus* cells elongate during the cell cycle

**Figure 1:**
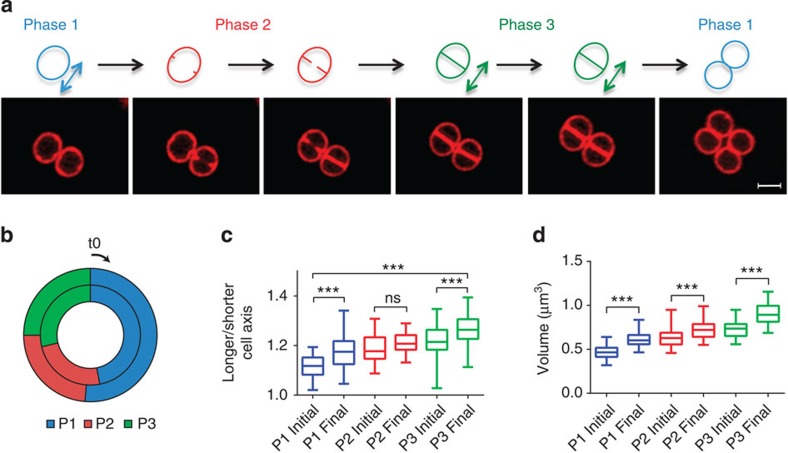
Morphological changes during the cell cycle of *Staphylococcus aureus.* (**a**) *S. aureus* COL cells were stained with membrane dye Nile Red and imaged by SR-SIM for 60 min, at 3 min intervals. Phase 1 (P1) cells have recently divided and have not initiated synthesis of the septum. Phase 2 (P2) cells are undergoing septum synthesis. Phase 3 (P3) cells have a complete septum and are going to split into two daughter cells. In the schematic representation of the cell cycle, coloured arrows indicate periods of cell elongation. Cells in the first panel are finishing Phase 1. Following panels correspond to 6 min intervals. Scale bar, 1 μm. (**b**) Duration of each phase of the cell cycle (represented as a fraction of the duplication time) was measured in individual cells imaged by SR-SIM (i) while growing at room temperature on agarose slides containing growth medium and followed for one phase only, to minimize photodamage effects (inner circumference); (ii) after growing in liquid culture, at 37 °C, and placed on the microscopy slide just before imaging (outer circumference). (**c**) Elongation of *S. aureus* cells during the cell cycle was evaluated by calculating the ratio of the longer to the shorter axes of each cell. *S. aureus* cells initiate the cell cycle with a shape close to a sphere and elongate during P1 and P3 (*P*≤0.001). No statistically significant elongation occurred during Phase 2 (*P*>0.05). *n*=40 cells for each phase. (**d**) Cell volume was measured at the beginning and at the end of each phase of the cell cycle. Cells increased their volume continuously throughout the cell cycle, from an average volume 0.47±0.07 μm^3^ at the beginning of P1 to an average volume 0.91±0.12 μm^3^ at the end of P3. *n*=40 for each phase. Data in (**c**,**d**) were collected from two independent experiments and are represented as box-and-whisker plots where boxes correspond to the first to third quartiles, lines inside the boxes indicate the median and ends of whiskers represent the minimum and maximum of all data. Statistical analysis was performed using the unpaired *t* test (***P<0.001; ns, not significant).

To follow morphology dynamics during the cell cycle of *S. aureus*, cells of type strain COL were labelled with the membrane dye Nile Red, placed on an agarose pad containing growth medium and imaged during growth at room temperature by Super-Resolution Structured Illumination Microscopy (SR-SIM; Fig. 1a and Supplementary Movie 1). During the initial phase of the cell cycle (here referred to as Phase 1), before initiation of division septum formation, cells were approximately spherical in shape, as determined by calculating the ratio between the longer axis of the cell (perpendicular to the division septum) and the shorter axis (coincident with the septum) and then became slightly elongated (Fig. 1c, Supplementary Fig. 1 and Supplementary Table 1). In a second stage (Phase 2), cells initiated and completed the synthesis of the division septum and did not significantly elongate. Finally, during the last stage of the cell cycle (Phase 3), cells with a complete septum elongated further, before splitting into two daughter cells. Splitting was accompanied by fast reshaping of the flat septum to become approximately one hemisphere of each daughter cell.

Although we were able to observe cells undergoing complete cell cycles by SR-SIM imaging, we noticed a delay in growth, when compared with cells on the same slide that were not exposed to laser light. Photodamage is cumulative and therefore could cause an increasing bias towards longer duration of growth phases over the course of the experiment. To overcome this limitation, all quantitative analyses were only performed on the first complete growth phase of each cell (that is, in cells observed finishing the previous phase and starting the next phase), and not on the entire cell cycle. Using this approach we measured the duration of each growth phase of the *S. aureus* cell cycle (Fig. 1b). Cells spent approximately half of the cell cycle in Phase 1 (47±9%), with the other half being spent in septum synthesis (Phase 2, 24±7%) and the final elongation step (Phase 3, 29±6%), with a cell cycle duration of 66±9 min. Because the cells analysed by this method were growing at room temperature on growth medium containing agarose pads, on the microscope stage, we independently confirmed the length of each phase by growing *S. aureus* cells in liquid culture with aeration at 37 °C, labelling cell membranes and immediately observing them by SR-SIM. The percentage of cells observed in each phase should be proportional to the fraction of the cell cycle spent in that stage. Figure 1b shows that similar results for the duration of Phases 1–3 were obtained by both methods.

**Figure 2:**
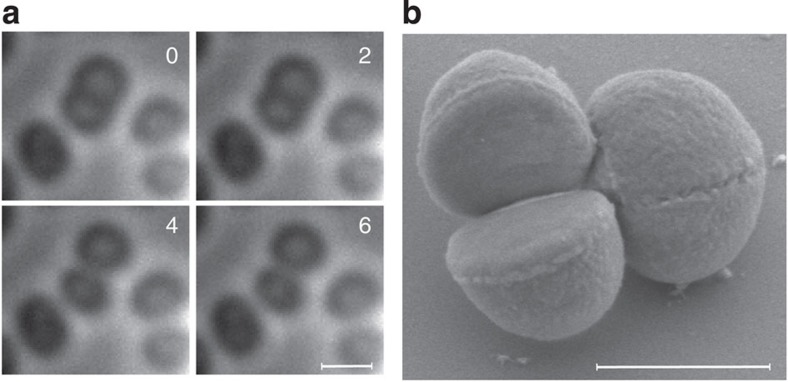
Splitting of *S. aureus* cells occurs on the millisecond timescale. (**a**) Dividing *S. aureus* cells were imaged on an agarose pad, acquiring frames every 2 ms, showing that splitting of the mother cell occurs in <2 ms (between the 2 ms and the 4 ms frame). Time is indicated in milliseconds. Scale bar, 1 μm. (**b**) To capture intermediate stages in the splitting process, *S. aureus* cells were fixed with glutaraldehyde during growth and imaged by SEM. The cell on the left has just divided in two daughter cells. The previous division septum is seen as a smooth flat surface indicating that septum splitting and reshaping into a curved hemisphere are likely to be sequential events. Note that cells with flat septa were not as rare as could be expected given the speed of the splitting process. It is therefore possible that the cell treatment required for SEM stabilizes this stage. Scale bar, 750 nm.

Interestingly, at the timescale of this experiment, we never observed intermediate stages in the process of cell splitting and reshaping of the flat septum into a curved surface, implying that these events are likely to be extremely fast. To verify this assumption, we imaged cells, growing on an agarose pad, at 2 ms intervals (Fig. 2a and Supplementary Movie 2). *S. aureus* cells divided into two daughter cells in the interval between two acquisitions, indicating that splitting takes less than 2 ms (*n*=10). The spatial and temporal resolution of these images does not allow us to determine if (i) splitting and septum reshaping occur simultaneously, as one could expect if remodelling of the flat septum did not require any enzymatic activity and resulted solely from the increased turgor pressure imposed on the septum as it splits and becomes exposed to the external milieu or (ii) splitting and septum reshaping are two consecutive processes. We reasoned that if the latter hypothesis was correct, we should be able to capture intermediates in the division process in which the septum had already split but remained flat. For this purpose we fixed cells during exponential growth and imaged using Scanning Electron Microscopy (SEM; Fig. 2b). In agreement with previous data^9^, we observed recently divided cells in which the previous septum was seen as a smooth flat surface, indicating that septum splitting and reshaping are likely to be sequential events.

### Septum generates less than one hemisphere of daughter cells

**Figure 3:**
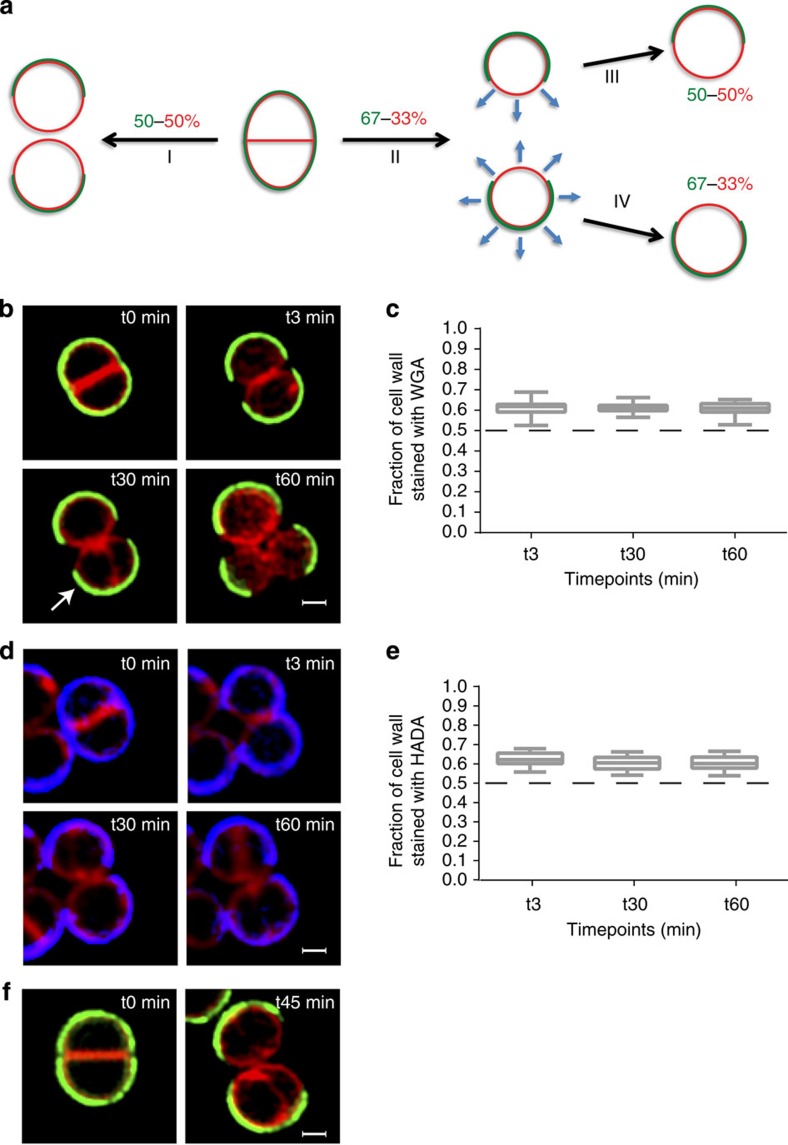
Asymmetrical inheritance of cell wall material during *S. aureus* cell division. (**a**) Schematic representation of the possible modes of division and growth of *S. aureus.* Peripheral cell wall of the mother cell is represented in green and cell membrane is represented in red. On division of the mother cell, the new cell wall material, derived from the previous septum, may constitute 50% of the surface of each daughter cell, if the surface area of the flat septum increases as it becomes curved (I), or 33%, if there is no increase in the surface area of septum material (II). In the latter case, the cell can then grow by peptidoglycan synthesis and/or autolysis either at the site of the new cell wall material only (III) or throughout the entire cell surface (IV). (**b**) *S. aureus* COL cells were labelled for 5 min with peripheral cell wall dye WGA-488 (green), washed and stained with membrane dye Nile Red. Cells were placed on an agarose pad and resumed growth at room temperature. On division, the old cell wall preserved the green WGA-488 signal. Cells were imaged by SR-SIM at 3, 30 and 60 min after splitting. The cell indicated by the arrow underwent a second round of division. (**c**) The fraction of WGA-488-labelled cell wall (green) relative to cell perimeter (red) remained above 0.60 during the cell cycle, *n*=30. No statistically significant variation was found (*P*>0.05). (**d**) An experiment equivalent to that described in panel (**b**) was done using cell wall dye HADA instead of WGA-488. (**e**) The fraction of cell wall labelled with HADA (blue) relative to the cell perimeter (red), decreased slightly during the first 30 min but remained above 0.60 during the cell cycle, *n*=30. (*P*=0.03). (**f**) The cell wall of *S. ureae* was labelled with WGA-488, washed, labelled with Nile Red and imaged by SR-SIM. Panels show the same cell before and after division, indicating that old cell wall material (green) constitutes more than half of the daughter cells surface. Contrast of individual channels was adjusted on merged images. Scale bars, 0.5 μm. Data in (**c**,**e**) are represented as box-and-whisker plots where boxes correspond to the first to third quartiles, lines inside the boxes indicate the median and ends of whiskers represent the minimum and maximum of all data. Statistical analysis was performed using one-way analysis of variance.

Previous descriptions of the *S. aureus* cell cycle suggested that cell wall synthesis occurred mainly, if not only, at the division septum^5^. Furthermore, it was assumed that the ‘new’ cell wall material, from the septum of the mother cell, formed one complete hemisphere of each of the daughter cells, while the other hemisphere was made of ‘old’ cell wall material originating from the peripheral wall of the mother cell. However, we observed that just before splitting, at the end of Phase 3, the mother cell has an ellipsoid shape, with a semi-major axis of 0.70±0.04 μm (perpendicular to the septum) and two semi-minor axes of 0.55±0.03 μm (equivalent to the radius of the septum) resulting in a septal surface area of 0.95 μm^2^. After splitting, at the beginning of Phase 1, each daughter cell has semi-major and semi-minor axes of 0.52±0.03 μm and 0.46±0.03 μm, respectively. Therefore, the total surface area of each daughter cell is ∼2.89 μm^2^ (see Methods section for Knud Thomsen approximation used for calculations), of which 0.95 μm^2^, or ∼33%, should be composed of new cell wall material, originating from the septum of the mother cell (in the absence of septum expansion). The remaining ∼67% of the cell surface should be composed of ‘old’ cell wall material, originating from one hemisphere of the mother cell, that is, the distribution of old and new cell wall material should not be 50% of each, as previously assumed^3^,^5^,^6^ (Fig. 3a).

To determine whether the mode of *S. aureus* growth was consistent with these assumptions, cells were labelled with a green fluorescent derivative of wheat germ agglutinin (WGA-488), a lectin that labels *N*-acetylglucosamine residues present in peptidoglycan and in wall teichoic acids. Importantly, WGA labels the cell wall present at the cell surface (peripheral wall) but does not diffuse into (and therefore does not label) the division septum. Cells were next washed to remove unbound WGA-488, and stained with the membrane dye Nile Red, which diffuses through the septum and therefore labels the entire membrane of the cell. Finally, cells were placed on an agarose pad containing growth medium and imaged by SR-SIM during growth (Fig. 3b). Cells whose surface was completely labelled by WGA-488 were selected for analysis, and both the perimeter of each cell as well as the fraction of the cell surface made of old cell wall (WGA-488 labelled) was measured. In agreement with our model, we found that after splitting of the mother cell, each daughter cell had 61.3±3.3% (*n*=30) of its surface labelled by WGA-488, that is, made of old cell wall, clearly showing that the flat septum, even after reshaping, does not contribute to half of the cell surface (Fig. 3c).

These results were confirmed in a second experiment, similar to the one described above, in which cells were labelled with Hydroxycoumarin-amino-D-alanine (HADA) instead of WGA-488. HADA is a blue fluorescent derivative of 3-amino-D-alanine that can be incorporated in the pentapeptide chain of peptidoglycan^10^. Selected cells whose entire surface was labelled with HADA at time zero were followed during growth for 60 min (Fig. 3d). Similar to the results obtained with WGA-488 labelling, on splitting of the mother cell 62.5±3.4% of the surface of each daughter cell was labelled with HADA.

**Figure 4:**
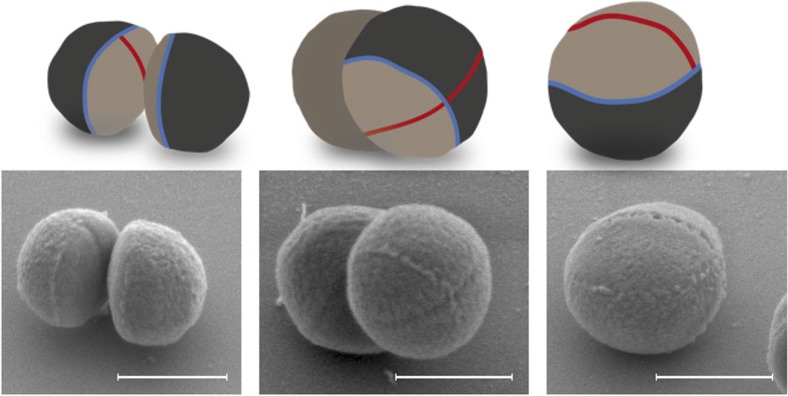
Scars of previous divisions do not mark cell quadrants. SEM images of *S. aureus* COL cells showing an asymmetric scar (blue line in scheme) corresponding to the previous division site and a fissure located in the middle of the cell (red line), presumably corresponding to the next division site. The new cell wall (light brown), resulting from septal material from the mother cell, which has a smooth surface immediately after division (first panel), occupies less than half of the total surface. Scale bars, 600 nm.

Careful inspection of SEM images of *S. aureus* cells confirms that the ‘old’ cell wall constitutes more than 50% of the surface of each daughter cell (Fig. 4). Cell wall material that is at least one generation old has been described as having an irregular, rough appearance, while newly synthesized cell wall, resulting directly from the septum of the mother cell, has a smoother appearance with concentric rings^3^,^11^, with a clear border separating the two types of cell surface^12^. These borders, seen in Fig. 4 and denoted in blue in the schematic representation of the cells, are clearly not placed at mid cell, reinforcing the idea that more than half of the surface of *S. aureus* cells is at least one generation old.

We then asked if this mode of division of spherical cells, in which the division septum of the mother cell generates less than one hemisphere of each daughter cells, was restricted to *S. aureus* or common to other cocci. Labelling of peripheral cell walls in *Sporosarcina ureae* with WGA-488 showed that division of spherical cells from this organism also results in daughter cells with ∼60% of the surface made of ‘old’ cell wall (Fig. 3f).

### *S. aureus* grows by remodelling the entire cell surface

The fact that cell wall synthesis in *Staphylococci* occurred mainly, if not exclusively, at the division septum^5^, led to the suggestion that *S. aureus* does not enlarge during the cell cycle and that the conversion of the flat division septum into one hemisphere of each daughter cell accounted for the doubling in volume required for cell division to take place^3^,^5^,^6^. Importantly, the diffraction-limited resolution of conventional light microscopy (∼250 nm)^13^,^14^ impaired, until now, detection of size variations in the range of those required for the doubling of *S. aureus* cell volume (see Discussion section). These variations can, however, be observed by SR-SIM, which improves the lateral (xy) resolution to ∼110 nm^15^. Using this technique, we observed that staphylococcal cells increased in volume during the entire cell cycle, from a volume of 0.47±0.07 μm^3^ at the beginning of Phase 1 to a volume of 0.91±0.12 μm^3^ at the end of Phase 3 (Fig. 1d and Supplementary Table 2). If enlargement of *S. aureus* cells was mainly due to the remodelling of the flat septum into a hemisphere, the volume increase should be essentially restricted to the process of splitting and reshaping of the septum. On the contrary, we have observed that there is no significant increase in volume when staphylococcal cells divide, that is, each of the two daughter cells has approximately half of the volume of the Phase 3 mother cell (Fig. 1d). Furthermore, the largest and fastest increase in volume occurs during Phase 3 (Δvolume=0.16±0.07 μm^3^ at a rate of 0.009±0.003 μm^3^ min^−1^, Supplementary Table 2), during which there is no reshaping of the flat septum, clearly indicating that other mechanisms must be involved with the enlargement of *S. aureus* cells.

Next, we wondered if enlargement of cells occurred via remodelling only of the material derived from the previous septum or via remodelling of the entire cell surface (Fig. 3a). In the former case, the percentage of ‘new’ cell wall should increase during the course of the cell cycle, eventually reaching half of the total cell surface, while in the latter case the ratio of new to old cell wall observed immediately after splitting of the mother cell (∼39% to ∼61%, respectively, Fig. 3b) should be maintained over the cell cycle. Therefore, following the growth of cells in which the ‘old’ cell wall was labelled with WGA-488 should allow us to determine how *S. aureus* cells enlarge. The perimeter of WGA-488-labelled cells increased from 3.08±0.17 μm to 3.40±0.21 μm after 60 min on the slide, confirming that the cells were growing. Under these conditions, the fraction of cell wall labelled by WGA-488 remained at 60.6±3.2%, even in cells that were already initiating the next round of division (Fig. 3b,c). Similar results were obtained with HADA-labelled cells, in which the fraction of labelled cell wall remained at 60.5±3.8% after 60 min (Fig. 3d,e), indicating that cell enlargement was due to remodelling of the entire cell surface and not exclusively of the septal material.

### Cell enlargement needs peptidoglycan synthesis and autolysis

Enlargement of bacterial cells can occur via synthesis of new peptidoglycan, autolysis of old peptidoglycan or a combination of both processes. We have previously observed that peptidoglycan synthesis occurred mostly at the division septum^5^. Given that we have now shown that growth of staphylococcal cells occurs via remodelling of the entire cell surface, we considered the possibility that enlargement was mainly due to autolytic activity. However, autolysis without synthesis would lead to a thinning and/or increase in the porosity of the old peptidoglycan mesh over consecutive generations. Since ∼60% of old cell wall is transmitted to daughter cells in each cell division, staphylococcal cells would have regions of the surface a few generations old, which were necessarily submitted to the enlargement process multiple times. Thinning of these regions owing to peptidoglycan autolysis without synthesis could therefore endanger the integrity of the bacterial cell. Making use of newly available tools to study peptidoglycan synthesis and to image protein localization, we revisited the question regarding the localization of peptidoglycan synthesis in *S. aureus.*

**Figure 5:**
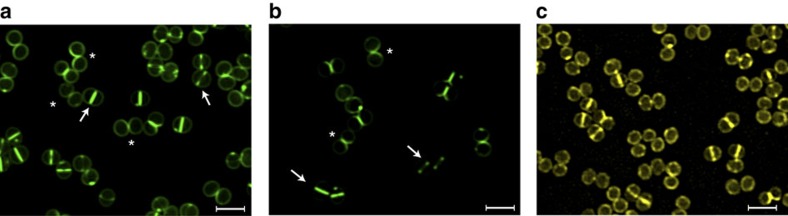
PBP4 is present at the division septum and peripheral cell wall. (**a**) *S. aureus* COL cells were labelled for 20 min with NADA, a fluorescent derivative of 3-amino-D-alanine that can be incorporated in the pentapeptide chain of peptidoglycan, and imaged by SR-SIM. Cells with partial (Phase 2) or complete (Phase 3) septa (arrows) showed labelling mostly at the septum, although some labelling could also be observed at the peripheral cell surface. Newly split cells and cells without septa (Phase 1) showed NADA incorporation around the cell surface (asterisks). (**b**) In a COLΔ*pbpD* strain, lacking PBP4, peripheral NADA incorporation was virtually absent both in cells undergoing septation (arrows) and in newly split cells (asterisks), indicating that PBP4 is responsible for the majority of peripheral NADA incorporation. (**c**) Localization of a YFP-tagged derivative of PBP4 in *S. aureus* COL cells by SR-SIM showed that although this protein was mostly localized at the septum, peripheral protein localization could also be observed in every cell, regardless of its cell cycle stage. Scale bars, 2 μm.

*S. aureus* cells were labelled with Nitrobenzofurazan-amino-D-alanine (NADA), a green fluorescent derivative of 3-amino-D-alanine that can be incorporated by PBPs in the pentapeptide chain of peptidoglycan^10^. Cells in growth Phases 2 and 3, that is, cells with partial or complete septa, showed labelling mostly at the septum, in agreement with previous reports showing that the majority of PBP activity takes place at the septum^4^,^5^,^16^. However, peripheral signal was also observable (Fig. 5a). Labelling around the entire cell surface was even more noticeable in Phase 1 cells, lacking a septum (Fig. 5a, cells labelled with asterisks), even when the labelling period was as short as 5 min (Supplementary Fig. 2).

We then asked which of the four native *S. aureus* PBPs had peripheral activity. Given that PBP1 and PBP2 are essential proteins in MSSA strains and therefore cannot be depleted^17^,^18^, *S. aureus* mutants lacking PBP3 and PBP4 were initially tested. COLΔ*pbpD* cells, lacking PBP4, were virtually devoid of NADA labelling away from the septum (Fig. 5b and Supplementary Fig. 3b), showing that this protein is the main enzyme responsible for the peripheral signal. Complementation of COLΔ*pbpD* with a plasmid-encoded PBP4 resulted in recovery of the peripheral signal (Supplementary Fig. 3c,d). A comparison of the ratio of the septal versus peripheral fluorescence signal, obtained by conventional epifluorescence microscopy, in the COL parental strain (7.91±0.42) and COLΔ*pbpD* (18.7±0.76) confirmed that incorporation of D-alanine-labelled analogues was essentially absent from the peripheral wall of the mutant lacking PBP4 (Supplementary Fig. 3e). In agreement, localization of a fluorescent derivative of PBP4 by SR-SIM showed that although this protein is mainly localized at the septum, as we have previously reported^19^, peripheral fluorescent signal could also be observed (Fig. 5c and Supplementary Fig. 3f). Quantification by PhotoActivated Localization Microscopy (PALM) showed that 26±11% of the total PBP4 molecules (fused to photoactivatable mCherry) in Phase 3 cells are present at the peripheral membrane (Supplementary Fig. 3g).

**Figure 6:**
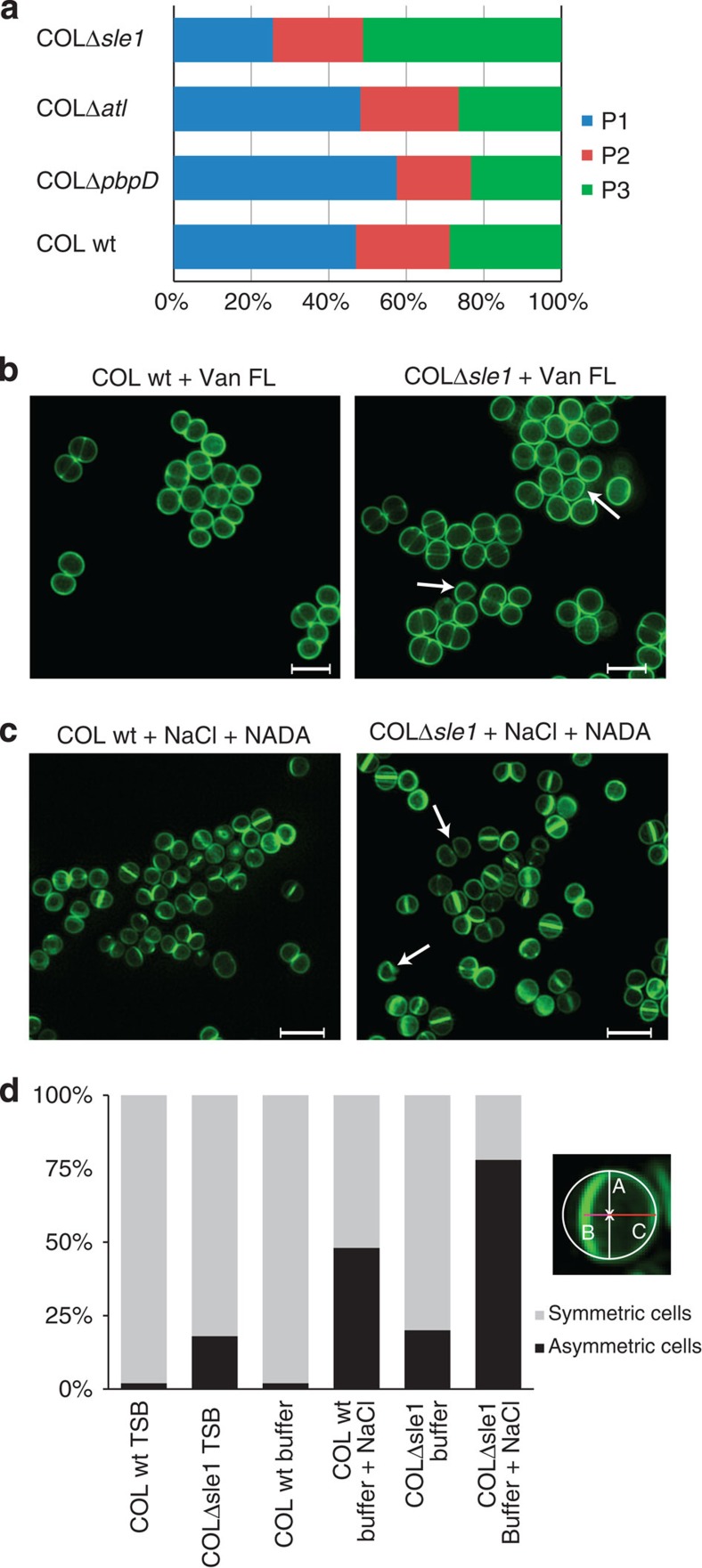
Effect of impaired peptidoglycan synthesis or autolysis on the cell cycle and morphology of *S. aureus.* (**a**) Duration of phases of the cell cycle of *S. aureus* mutants lacking peptidoglycan synthesis enzyme PBP4 (COLΔ*pbpD*, total duration of cell cycle 73±10 min) or autolysins Atl (COLΔ*atl*, 82±8 min) and Sle1 (COLΔ*sle1*, 86±15 min), compared with the parental strain COL (66±9 min). Cells (*n*=40 for each phase) were growing in solid medium and only cells that completed at least one growth phase during the time of the experiment were included in the analysis. Approximately 50% of COLΔ*pbpD* cells that initiated Phase 1 did not complete it (versus 20% of COL cells) and were not included in the analysis. (**b**) The cell wall of COL and COLΔ*sle1* was stained with Van-FL and imaged by SR-SIM. Asymmetric cells with a shape close to a ‘D’ (white arrows) were observed, indicating that reshaping of the flat septum into a curved hemisphere following division was impaired in this mutant. (**c**) Exponentially growing COL and COLΔ*sle1* cells were stained with NADA, incubated for 15 min in saturating concentration of NaCl, placed on an agarose pad containing the same salt concentration and imaged by SR-SIM. Cells with a shape close to a ‘D’ (examples indicated by white arrows) were observed at higher frequency in the mutant lacking Sle1 (right) than in the parental strain COL (left). Scale bars, 2 μm. (**d**) Symmetry of cells depicted in panels **b** and **c** was assessed by fitting an ellipse to the cells (*n*=50 for each class) and defining its centre (X) as the middle point of the shorter axis (A). The distances from X to new peripheral cell wall (B) and old peripheral cell wall (C) were calculated. Cells were considered as asymmetric (black bars) if the ratio C/B was more than 1.33 and symmetric (grey bars) when this ratio was ≤1.33. wt, wild type.

Furthermore, the duration of Phase 1 is longer in COLΔ*pbpD* than in the parental strain COL (Fig. 6a) and the cell volume is smaller (Supplementary Table 2), reinforcing the hypothesis that PBP4 is one of the proteins that have a role in peripheral peptidoglycan synthesis during this growth phase.

In contrast, cell elongation is unaffected in COLΔ*pbpD* cells (Supplementary Table 1), suggesting that additional proteins have a role in cell growth. We therefore evaluated the role of two major autolysins, Atl (ref. 7) and Sle1 (ref. 20) in cell enlargement and elongation, as well as in septum remodelling, by deleting chromosomal *atl* or *sle1* genes of strain COL. The lack of Atl amidase and glucosaminidase activities led to larger cells that are less elongated when compared with the parental strain COL (Supplementary Tables 1 and 2), indicating that Atl autolytic activity is involved not only in cell separation, as previously shown^8^,^9^, but also in cell size homoeostasis and shape maintenance. COLΔ*sle1* cells remain elongated throughout the entire cell cycle, which could result from increased stiffness of peptidoglycan in the absence of Sle1 amidase activity, possibly impairing correct reshaping of the cell following division. Furthermore, these cells have a longer Phase 3 than the parental strain COL (Fig. 6a and Supplementary Fig. 4), suggesting that cell splitting is impaired in this mutant.

Interestingly, we have observed the appearance, albeit at low frequency, of cells with the shape of the letter ‘D’ in cultures of COLΔ*sle1* (Fig. 6b). These cells seem to be impaired in the process of reshaping of the flat septum, generated immediately after the splitting of the mother cell, into a curved hemisphere. D-shaped cells were never observed in the parental strain COL by SR-SIM. This phenotype seems to be a consequence of defects in specific autolytic enzymes whose activity is required for the reshaping of the flat septum, since asymmetric cells were also identified at higher frequencies than in the parental strains in (i) an *sle1* deletion mutant in the background of a different *S. aureus* strain, namely NCTC8325-4 (Supplementary Fig. 5b); (ii) an NCTC8325-4 mutant lacking LytM, an autolysin with glycylglycine endopeptidase activity^21^ (Supplementary Fig. 5c); and (iii) a COL mutant depleted for the two-component system WalK/WalR (also known as YycG/YycF), which positively controls autolytic activity^22^ (Supplementary Fig. 5e). Interestingly, although WalKR is essential in RN4220 (ref. 22), strain COL grows in its absence.

To determine if turgor pressure also had a role in driving the reshaping of the flat septum, we incubated COL and COLΔ*sle1* in buffer containing saturating NaCl concentration. The decreased turgor pressure led to higher frequency of ‘D’ shaped or asymmetric cells both in COLΔ*sle1* and in parental strain COL, albeit to lower levels in the parental strain (Fig. 6d). Strain COLΔ*sle1* complemented with a plasmid-encoded Sle1 behaved as the parental strain COL (Supplementary Fig. 6). These results suggest that the division septum requires the action of both autolytic enzymes and turgor pressure to change from a flat to a curved surface, although we cannot exclude the possibility that the effect of NaCl is due to an impact on the activity of autolysins.

## Discussion

Despite its importance as a clinical pathogen, the small size of *S. aureus* cells has impaired a detailed analysis of the morphological changes occurring during its cell cycle, given that size variations required to double the cell volume are close to the limit of resolution of conventional optical microscopy: *S. aureus* cells are ∼1 μm in diameter (or 0.52 μm^3^ in volume). Approximating the cell shape to a sphere, an increase of the diameter to 1.26 μm is sufficient to double the cell volume. However, this 260 nm increase is close to the diffraction-limited resolution of conventional optical microscopy, which is ∼250 μm for most biocompatible fluorophores^13^,^14^.

The current model for *S. aureus* cell division postulates that cells do not significantly enlarge during the cell cycle and that the major variation in cell volume occurs on splitting of the mother cell, when the flat septum is reshaped into a curved surface to generate one hemisphere of each daughter cell. This model was based on the fact that both incorporation of new peptidoglycan material and localization of the major peptidoglycan synthesis enzymes, the PBPs, occurred mostly at the septum^5^,^16^,^17^,^19^,^23^, two observations that still hold true. However, here, by fast imaging of dividing *S. aureus* cells, we show that reshaping of the division septum is an extremely rapid process, on the timescale of <2 ms, and therefore not compatible with a duplication of cellular volume and the consequent abrupt changes in concentrations of cellular contents. It is therefore more likely that, similar to other bacteria, the volume of *S. aureus* cells gradually increases over the cell cycle, but these size variations are too small to be observable by conventional light microscopy.

**Figure 7:**
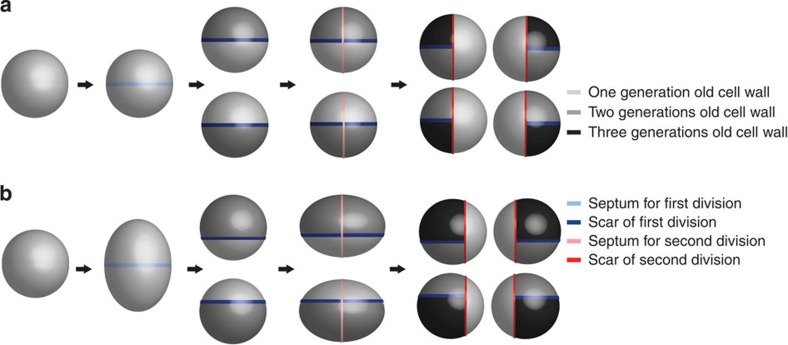
Comparison of two models for *S. aureus* growth and division. (**a**) Previous model which assumed that *S. aureus* cells remained approximately spherical over the cell cycle and that, on division, the cell wall material from the septum of the mother cell increased in cell surface area to constitute half (one hemisphere made of new cell wall) of the cell surface of each daughter cell. As a consequence, scars of previous divisions divided the cell in quadrants and formed T-junctions at the cell poles, which could be used as topological cues to direct cellular processes such as chromosome segregation. These scars were proposed to encode epigenetic information that could be used by *S. aureus* to determine orthogonal placement of division septum. (**b**) In the model proposed based on this work, *S. aureus* cells are approximately spherical at the beginning of the cell cycle and elongate as the cell cycle progresses. On division, there is no increase in the surface area of the previous septum, which becomes ∼33% of the surface area of each daughter cell. This asymmetry in the regions composed of new and old cell wall results in scars of previous divisions that do not divide cells in quadrants. Consequently, T-junctions of scars of two previous divisions are not located at the cell poles. Two consecutive divisions in orthogonal planes are depicted in panels (**a**,**b**).

Recent advances in fluorescence microscopy, specifically the introduction of super-resolution techniques, now allow an unprecedented level of detail in the analysis of bacterial cell morphology. Making use of SR-SIM, with a lateral resolution of ∼110 nm (ref. 15), we measured variations of the volume of *S. aureus* cells and concluded that (i) cell volume increases gradually over the entire cell cycle and (ii) there is no substantial increase in cell volume due to reshaping of the flat septum, given that on splitting of a mother cell, each newly generated daughter cell has approximately half of the mother cell’s volume. Furthermore, and contrary to previous assumptions, the cell wall material resulting from the division septum of the mother cell (new cell wall) does not constitute half of the cell surface of each daughter cell, but ∼33% (Fig. 7). Taken together, these observations suggest that other mechanisms besides septum reshaping are responsible for cell growth in *S. aureus.*

On division of the mother cell, *S. aureus* cells could grow by (i) remodelling and enlarging only the new cell wall material or (ii) remodelling the entire cell surface. In the first case, the surface area corresponding to new cell wall material would be expected to expand from 33% and eventually constitute half of the cell surface, while in the second case the 33/67 ratio of new/old cell wall would be maintained during the entire cell cycle. We have labelled peripheral cell wall with a lectin (WGA-488) as well as a fluorescent derivative of D-alanine (HADA) and confirmed that the latter hypothesis is correct.

Cell enlargement can occur by synthesis of new peptidoglycan material, autolysis of old peptidoglycan or a combination of both processes. The fact that the entire cell surface is enlarged during growth, but different sections of the surface vary in age, seems incompatible with the notion that peptidoglycan synthesis or autolysis alone are responsible for the increase in cell surface. If that were the case, then regions with different ages of the cell wall would have different thickness and/or porosity. By transmission electron microscopy, *S. aureus* peripheral cell wall seems homogeneous in thickness^24^. The porosity of new and old cell wall may be different, as the former shows a series of concentric rings while the latter appears as a network of fibres with a large number of empty spaces between them, perhaps resulting from autolytic activity^11^,^25^. However, if this autolytic activity would occur across the entire cell surface multiple times, that is, over several generations, in the absence of peptidoglycan synthesis, holes in older regions of the cell surface would presumably become larger, possibly endangering the integrity of the cell.

It therefore seems likely that peptidoglycan synthesis has to occur across the cell surface, concomitantly with autolysis. In fact, we were able to detect peripheral PBP activity through the incorporation of fluorescent derivatives of 3-amino-D-alanine^10^, mediated mostly by PBP4, in agreement with recent data by Gautam *et al*.^26^. It is possible that other PBPs also have a role in peripheral peptidoglycan synthesis, which is not detected in our assay given that PBP4 is the main responsible for incorporation of exogenous peptidoglycan synthesis probes^26^,^27^. It is important to note that these probes may be incorporated by PBPs in an exchange reaction that occurs outside the cell, and therefore do not necessarily reflect the sites of incorporation of lipid-linked peptidoglycan precursors. It is possible that PBP4 activity is required, not (only) to incorporate new peptidoglycan precursor molecules, therefore increasing the amount of peptidoglycan material in the cell wall, but also to make new bonds in pre-existing material that was subjected to autolytic activity during cell expansion leading to an increased mechanical strength. In fact PBP4 was shown to be responsible for the very high levels of peptidoglycan crosslinking characteristic of *S. aureus*^28^,^29^ and required to increase the stiffness of the cell envelope^30^.

Finally, we have also pondered over the question of the extremely fast process of septum reshaping into a curved hemisphere, namely if it was a purely mechanical process or if it required enzymatic activity. Previous reports using atomic force microscopy imaging have shown the presence of perforation holes around the bacterial circumference coincident with the outer edge of the division septum. These holes become larger, merge and form longer nicks in the peripheral wall as splitting of the septum proceeds^25^. These holes are most likely the result of activity of autolysins such as Atl, a protein required for cell separation, which localizes in rings at the division site^8^,^9^. Interestingly, Atl localizes exclusively at the external edge of the septum, not across the entire septal surface^9^, suggesting that its activity is only required at the initial steps of splitting. This would be in agreement with the suggestion by Matias and Beveridge that the septum of *S. aureus* has a middle zone that separates two adjacent septal cross walls^31^. Once the outer edge of the septum is cleaved by autolysins, cell separation could proceed through a merely mechanical process driven by turgor pressure on the two separate halves of the cross-wall, as no additional cleavage of peptidoglycan bonds would be required across the septum. The time required for splitting, which we have now shown to occur in <2 ms, does indeed suggest a mechanical process, probably driven by turgor pressure, in agreement with our data showing that septum reshaping is impaired in high osmolarity conditions and with recent data published while this manuscript was under revision^32^. However, we have also shown that autolysins such as Sle1 and LytM have a role in reshaping of the flat septum, given that mutants lacking one of these proteins, particularly when exposed to medium with high osmolarity, generate daughter cells in which the previous septum remains flatter. Nevertheless, we cannot determine if Sle1 and LytM activities occur before or during splitting. Interestingly, Bailey *et al*.^33^ have recently shown that the new cell wall material in the flat septum is stiffer than the rest of the cell wall and becomes softer when fully expanded into a hemisphere. Therefore, these authors suggest that reshaping of the flat septum cannot occur only through stretching (as this would require the flat septum to be thicker but softer than the rest of the cell wall), but instead requires partial degradation of the peptidoglycan by autolytic enzymes^33^.

The increase in resolution resulting from advances in super-resolution microscopy has allowed us to completely redefine the cell cycle of *S. aureus*, one of the most relevant bacterial pathogens, with important implications for current models of cell division in cocci. *S. aureus* cells divide in three orthogonal planes over three consecutive division cycles^1^,^2^, raising the question of how do the cells retain ‘memory’ of the two previous planes of division, to define a third, perpendicular, division plane. Scars of previous divisions have been proposed to contain epigenetic information regarding the previous planes of division^3^. Junctions of these scars at the cell poles could be used as topological cues to drive chromosome segregation in an axis perpendicular to the next division plane^34^. The process of nucleoid occlusion, which prevents assembly of the divisome over the chromosome, would then restrict the localization of the next division plane to a plane orthogonal to the two previous ones^34^. However, these models assume that scars of previous division planes are placed precisely at the centre of the cell (similar to the equator or meridians of earth, Fig. 7a), and therefore cross each other at poles of the cells. Our observation that lines dividing the *S. aureus* cell surface containing old/new cell wall material do not divide the cell in two equal parts (Fig. 7b), reopens the question of how *staphylococci* divide in orthogonal planes. Our findings may also be extended to other cocci that divide in orthogonal planes, such as *Sporosarcina*, and thus our observations described for *S. aureus* should have implications for the global understanding of cell division in other coccal bacteria.

## Methods

### Bacterial growth conditions

Strains and plasmids used in this study are listed in Supplementary Table 3. *S. aureus* strains were grown in tryptic soy broth (TSB, Difco) with aeration at 37 °C or on tryptic soy agar (Difco) at 30 or 37 °C. For microscopy experiments, overnight cultures of *S. aureus* strains were diluted 1:200 in fresh TSB medium and allowed to grow at 37 °C until an OD_600 nm_ of ∼0.5. Cells were then collected and resuspended in the same medium. *E. coli* and *S. ureae* strains were grown in Luria–Bertani broth (Difco) with aeration, or Luria–Bertani agar (Difco) at 37 or 30 °C, respectively. Antibiotics ampicillin and erythromycin were added to the media at a final concentration of 100 and 10 μg ml^−1^, respectively, when necessary. 5-Bromo-4-chloro-3-indolyl β-D-galactopyranoside (X-gal) was used at 100 μg ml^−1^. Expression of PBP4 from plasmid pBCBPM115 was induced with cadmium chloride (1 μM). Expression of Sle1 from plasmid pSle1 was induced with IPTG (1 mM) in the presence of 10 μg ml^−1^ chloramphenicol.

### Construction of *S. aureus* mutants

The COLΔ*atl*, COLΔ*sle1* and NCTCΔ*lytM* null mutants (Supplementary Table 3) were constructed using the pMAD vector^35^ containing the upstream and downstream regions of each gene of interest, to allow recombination and integration of the plasmids into the chromosome, followed by their excision with the genes to be deleted. Briefly, upstream and downstream regions of *sle1* and *lytM* were amplified by PCR, using primers P1_sle1/P2_sle1 and P3_sle1/P4_sle1, P1_lytM/P2_lytM and P3_lytM/P4_lytM, respectively (Supplementary Table 4). The PCR fragments encoding the upstream and downstream regions of each gene were joined by overlap PCR using the pairs of primers P1_Sle1/P4_Sle1 and P1_lytM and P4_lytM. The resulting fragments were digested with *Nco*I and *Bgl*II (Fermentas) and cloned into the pMAD vector, giving plasmids pΔ*sle1* and pΔ*lytM*, which were propagated in *E. coli* DC10B and whose inserts were sequenced. The plasmids, as well as pΔ*atl*^36^, were then electroporated into *S. aureus* RN4220 strain at 30 °C, using erythromycin and X-gal selection, and transduced into COL or NCTC8325-4 using phage 80α (ref. 37). The integration of the plasmids into the chromosome and their excision was done as previously described^23^. Gene deletions were confirmed by PCR and sequencing of the amplified fragment.

For complementation of COLΔ*sle1*, plasmid pBCBover was constructed by introducing a DNA fragment containing the P_*spac*_ promoter, a multiple cloning site and the *lacI* gene into the *S. aureus* replicative vector pGC2 (ref. 38). For that, two DNA fragments, one containing the P_*spac*_ coding sequence and HindIII, SmaI and XbaI restriction sites and a second one encompassing a BglII restriction site upstream of the *lacI* gene, were amplified from pDH88 plasmid^39^ by using, respectively, the pair of primers Pspac_pDH88_P9_XhoI_SalI/Pspac_pDH88_SpeI_P10 and Pspac_pDH88_SpeI_P11/Pspac-pDH88-P12-XhoI-EcoRI. These two PCR products were then joined in a second PCR, using primers Pspac-pDH88_P9_XhoI_SalI and Pspac-pDH88-P12-XhoI-EcoRI, to originate a 1,743 bp fragment in which a SpeI restriction site was introduced between XbaI and BglII restriction sites. The resulting fragment was restricted with SalI and EcoRI, cloned into the pGC2 vector and sequenced. The pSle1 plasmid was constructed by cloning the entire *sle1* coding sequence downstream of P_*spac*_ promoter in pBCBover. For that, a 1,024 bp DNA fragment containing *sle1* gene and its upstream ribosomal binding site was amplified from NCTC8325-4 genome using primers Sle1_FW_XmaI and Sle1_RV_XbaI, digested with SmaI and XbaI and cloned into pBCBover. The insert was then sequenced.

Both pBCBover and pSle1 plasmids were electroporated into RN4220 (selection with 10 μg ml^−1^ chloramphenicol) and subsequently transduced, using phage 80α, to strain COLΔ*sle1*, generating strains COLΔ*sle1*pBCBover and COLΔ*sle1*pSle1, respectively.

Construction of an *S. aureus* COL mutant with *yycFG* (also known as *walKR*) under the control of the IPTG-inducible P_*spac*_ promoter was done using the pMUTIN4 plasmid^40^. A 518-bp fragment containing the ribosome binding site and 5′ end of *yycF* (*walR*) was amplified using primers yycF_fw_EcoRI and yycF_stop_rv_BamHI. The DNA fragment was then digested with *Eco*RI and *Bam*HI, cloned into pMUTIN4 giving plasmid p*walKR*, which was propagated in *E. coli* DC10B and whose insert was sequenced. Plasmid p*walKR* was then electroporated into *S. aureus* RN4220 at 37 °C, using erythromycin selection, and transduced into COL using phage 80α. Integration of the plasmid occurred through a single crossover event, placing the *walKR* operon under the control of the P_*spac*_ promoter.

To study PBP4 localization in *S. aureus* NCTC8325-4 strain by PALM, we replaced the *pbpD* gene, encoding PBP4, for a gene encoding a photoactivatable derivative (*pbpD-PAmCherry*). For that purpose, *pbpD* was cloned fused to *PAmCherry1* (ref. 41) in the backbone of pCNX plasmid. The pCNX plasmid was constructed by substituting the *erm* erythromycin resistance cassette of the pCN51 plasmid^42^ by the *aphA-3* kanamycin cassette from pCN34 (ref. 42), using *ApaI* and *XhoI* restriction sites. To clone *pbpD-PAmCherry* into pCNX, a fragment encompassing a ribosomal binding site, the *pbpD* gene lacking the stop codon and seven codons encoding an amino-acid linker was amplified using NCTC8325-4 genomic DNA as template and primers P1pbp4pCNX and P2pbp4pCNX. A fragment encompassing *PAmCherry1* was amplified from the pBAD/HisB-PAmCherry1 plasmid^41^ using P3pbp4pCNX and P4pbp4pCNX primers. The two fragments were joined by overlap PCR, digested with *BamHI* and *EcoRI*, ligated into the pCNX plasmid generating plasmid pBCBRP007, whose insert was sequenced. We then replaced the *pbpD* gene from its native locus in the *S. aureus* genome by the *pbpD-PAmCherry* fusion. For this purpose two DNA fragments were amplified, one encompassing a truncated *pbpD* (last 999 bp), a sequence encoding a seven-amino-acid linker and *PAmCherry1* amplified from the pBCBRP007 plasmid using primers P1pMADpbp4PAmCherry and P2pMADpbp4PAmCherry and a second DNA fragment of 1,000 bp corresponding to the region downstream of *pbpD* amplified from the NCTC8325-4 genome using primers P3pMADpbp4PAmCherry and P4pMADpbp4PAmCherry. The two fragments were joined by overlap PCR using primers P1pMADpbp4PAmCherry and P4pMADpbp4PAmCherry, digested with *BamHI* and *XmaI* restriction enzymes and cloned into pMAD, generating pBCBRP008 plasmid. Correct sequence of the insert was confirmed and the pBCBRP008 plasmid was electroporated into RN4220 and subsequently transduced to NCTC8325-4 strain using phage 80α. Integration and excision of pBCBRP008 from the genome was performed as previously described^23^, colonies in which *pbpD* had been replaced by the *pbpD-PAmCherry* were identified by PCR and the strain was named NCTCΔ*pbpD::pbpD-PAmCherry*.

To complement COLΔ*pbpD* with a plasmid encoding PBP4, the 1.3 Kb *pbp4* gene was PCR amplified from NCTC8325-4 genomic DNA using primers PBP4pCadP1BamFW and pCADPBP4wt_EcoREV and cloned into the pCNX replicative plasmid, under the control of a cadmium inducible promoter, using *BamHI* and *EcoRI* sites, generating plasmid pBCBPM115. Plasmids pCNX and pBCBPM115 were electroporated into RN4220 and then transduced into COLΔ*pbpD*, giving rise to strains BCBPM120 (COLΔ*pbpD*pCNX) and BCBPM138 (COLΔ*pbpD*pPBP4).

### Scanning electron microscopy

Exponentially growing *S. aureus* COL cells^43^ were collected by centrifugation, resuspended in fixative solution (2.5% glutaraldehyde in 0.2 M sodium cacodylate buffer, pH 7.4), deposited on glass discs (Marienfeld) and kept for 1 week at 4 °C. The fixative solution was subsequently removed and the cells were washed three times with sodium cacodylate solution. The sample was progressively dehydrated by immersion in a graded series of ethanol (50–100%) and then mounted on aluminium stubs with carbon adhesive discs (Agarscientific). The sample was critical-point dried under CO_2_ and sputter coated with gold–palladium (Polaron SC7640) for 200 s at 10 mA. SEM observations were performed using secondary electron images (2 kV) with a Hitachi S4500 instrument at the Microscopy and Imaging Platform (Micalis, B2HM, Massy, France) of the INRA research centre of Jouy-en-Josas (France).

### Super-resolution structured illumination microscopy

SR-SIM imaging was performed using a Plan-Apochromat 63x/1.4 oil DIC M27 objective, in an Elyra PS.1 microscope (Zeiss). Images were acquired using either three or five grid rotations, with 34 μm grating period for the 561 nm laser (100 mW), 28 μm period for 488 nm laser (100 mW) and 23 μm period for 405 nm laser (50 mW). Images were acquired using a Pco.edge 5.5 camera and reconstructed using ZEN software (black edition, 2012, version 8.1.0.484) based on a structured illumination algorithm^44^, using synthetic, channel specific optical transfer functions and noise filter settings ranging from −6 to −8.

### Fast time-lapse imaging of *S. aureus*

A 2 μl aliquot of an exponentially growing culture of *S. aureus* was placed on an agarose pad in TSB. Cells were imaged at 2 ms intervals for a total time of 10 s, using a Pco.edge 5.5 camera and a Plan-Apochromat 100x /1.46 NA Oil DIC ELYRA objective in an Elyra PS.1 microscope (Zeiss).

### Labelling and imaging of *S. aureus*

For time-lapse experiments, *S. aureus* cells were incubated with the membrane dye Nile Red (Invitrogen) at a final concentration of 10 μg ml^−1^, for 5 min at 37 °C, with agitation (550 r.p.m.). Subsequently, the cells were placed on an agarose pad containing 50% TSB in phosphate buffer saline (PBS) and imaged during growth by SR-SIM. Image sets were acquired every 3 min, for a total period of 1 h, using 2% of 561 nm laser power and 50 ms exposure times. Measurements were performed on reconstructed super-resolution images using ZEN software.

To determine the fraction of old/new cell wall in *S. aureus*, cells were stained with either a wheat germ agglutinin Alexa Fluor 488 conjugate (WGA-488, Invitrogen) or fluorescent D-amino-acid HADA^10^,^45^ at a final concentration of 2 μg ml^−1^ and 250 μM, respectively. The cells were incubated at 37 °C with agitation for 5 or 30 min for WGA-488 or HADA, respectively. Unbound dye was removed from the media by washing cells with TSB and cells were then incubated with Nile Red (10 μg ml^−1^) for 5 min at room temperature and placed on an agarose pad containing 50% TSB in PBS. Cells showing uniform WGA-488 or HADA staining were imaged by SR-SIM at 0, 3, 6, 30 and 60 min, using 2% 561 nm laser with 50 ms exposure for Nile Red, 3% 488 nm laser with 50 ms exposure for WGA-488 and 20% 405 nm laser with 100 ms exposure for HADA.

To evaluate localization of peptidoglycan synthesis activity, *S. aureus* cells grown to an OD_600 nm_ of ∼0.5 were labelled with fluorescent D-amino-acid NADA (250 μM) for 20 min at 37 °C, 550 r.p.m. Cells were then washed with PBS, placed on an agarose pad and visualized by SR-SIM, using 10% 488 nm laser, 100 ms exposure. For pulse labelling experiments, *S. aureus* cells were incubated with either HADA or its L-enantiomer HALA^10^ for 5 min at 37 °C, 550 r.p.m. and then imaged as described above.

*S. aureus* cell wall labelling with vancomycin was performed by incubating the cells for 2 min at room temperature with a mixture containing equal amounts of vancomycin (Sigma) and a BODIPY FL conjugate of vancomycin (Van-FL, Molecular Probes) to a final concentration of 0.8 μg ml^−1^. Cells were visualized by SR-SIM, using 2% 488 nm laser, 100 ms exposure.

### Labelling and imaging of *S. ureae*

To determine the fraction of old/new cell wall in *S. ureae* by time-lapse microscopy, cells from cultures at OD_600_∼0.5 were stained with WGA-488 at a final concentration of 6 μg ml^−1^. The cells were incubated at 30 °C with agitation for 10 min. Unbound dye was removed from the medium by washing cells with Luria–Bertani broth. Cells were then incubated with Nile Red (10 μg ml^−1^) for 5 min at room temperature and placed on an agarose pad containing 50% Luria–Bertani broth in PBS. Cells showing uniform WGA-488 labelling were imaged by SR-SIM at 0, 15, 30, 45 and 60 min, using 2% 561 nm laser with 50 ms exposure for Nile Red, 3% 488 nm laser with 50 ms exposure for WGA-488.

### Photoactivated localization microscopy

To observe PBP4-PAmCherry single molecules by PALM, an overnight culture of NCTCΔ*pbpD::pbpD-mCherryPA* was diluted 1/200 in TSB and incubated at 37 °C. At mid-exponential phase (OD_600 nm_ 0.6), 1 ml of culture was harvested by centrifugation and washed with 1 ml of PBS. Cells were resuspended in 20 μl of PBS and 1 μl was placed on a thin layer of 1.2% agarose in PBS mounted on a gene frame (Thermo Scientific). Image sequences for PALM analysis were obtained using a Zeiss Elyra PS.1 microscope with a × 100 1.46 NA objective, additional magnification of × 1.6, equipped with an electron multiplying charge-coupled device camera (Andor—iXon DU897) using the Zeiss ZEN software. For an initial bleach of auto-fluorescent molecules and unwanted preactivated fluorophores, the sample was first imaged with a 561 nm laser at ∼0.76 kW cm^−2^ and 33 ms exposure for 2,000 frames. After bleaching, the continuous activation and imaging of PAmCherry molecules was performed for 8,000 frames by simultaneously irradiating the sample with a 405 nm laser at ∼1.9 W cm^−2^ and a 561 nm laser at ∼0.76 kW cm^−2^, using 33 ms of exposure time. To maintain a stable density of fluorophores photo activating in each frame and compensate the depletion of PAmCherry molecules during the course of the experiment, the intensity of the 405 nm laser was gradually increased until ∼38 W cm^−2^. Post-processing of the last 8,000 frames was performed using the ZEN software where an *xy* two-dimensional-Gaussian fit was applied to the individually resolvable subdiffraction molecules present in each frame (mask of 9 pixels with a minimum signal-to-noise ratio of 6).

### Hyperosmotic shock

COL and COLΔ*sle1* cells were grown in TSB until an OD_600 nm_ of ∼0.5 and then incubated for 30 min at 37 °C with agitation (700 r.p.m.) in the presence of NADA (250 μM). The cells were collected, washed once with 50 mM Tris-HCl buffer pH=7.5 containing a saturating concentration of NaCl (4.96 M) and incubated for 15 min at 37 °C. Cells were pelleted and placed on an agarose pad containing the same medium used to perform the hyperosmotic shock and imaged by SR-SIM.

### Calculation of cell dimensions

To calculate the volume of each cell, an ellipse was fitted to the border limits of the cellular membrane of Nile-Red-labelled cells, overlaying the membrane dye signal. Subsequently, the shorter and longer axes were measured, coinciding with the septum and the axis perpendicular to it, respectively. The volume of the cell was obtained by an approximation to the volume of a prolate spheroid (equation (1)) where *a* and *b* correspond to the longer and shorter semi-axes, respectively.




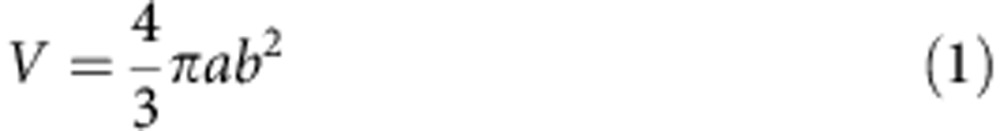




Cell surface area was calculated using the Knud Thomsen approximation^46^ (equation (2)) to calculate surface area of ellipsoids, where *a* corresponds to the longer semi-axis and *b* and *c* correspond to shorter semi-axes, which are identical in the case of *S. aureus* cells.




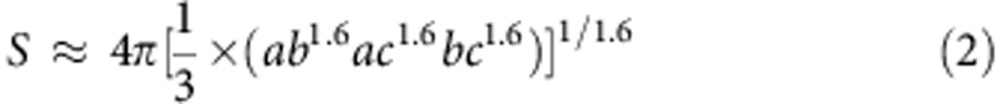




To evaluate cellular symmetry and identify ‘D’ shaped cells, cells in Phase 1 of the cell cycle were selected and an ellipse was fit to the cell borders corresponding to the old cell wall. The ellipse centre was defined as the middle point of the longer axis and the distances from this point (along a perpendicular axis) to new peripheral cell wall and old peripheral cell wall were calculated (see Fig. 6d). Symmetry was assessed by the ratio between the distance from the centre to the old cell wall and the distance from the centre to the new cell wall. A cell was considered as asymmetric when this ratio was more than 1.33, that is, when the distance from cell centre to the new cell wall was <75% of the distance to the old cell wall.

### Calculation of fluorescence ratio in NADA-labelled cells

NADA-labelled *S. aureus* cells were observed using a Zeiss Axio Observer microscope equipped with a Photometrics CoolSNAP HQ2 camera (Roper Scientific) and Metamorph 7.5 software (Molecular Devices). To quantitatively assess D-amino-acid incorporation, the fluorescence signal at the septum and at the peripheral cell wall was determined for 50 cells with fully formed septa. A fluorescence ratio for septal versus peripheral cell wall signals was determined as previously described^47^.

### Statistical analysis

Statistical analyses were done using GraphPad Prism 6 (GraphPad Software). Unpaired student’s *t*-tests were used to evaluate the differences in cellular volume and shape between cell cycle stages and between the initial and final stages of each phase, as well as to compare fluorescence ratios between peripheral and septal wall signal intensity. One-way analysis of variance was used to compare old/new cell wall fraction at different time points. *P* values ≤0.05 were considered as significant for all analysis performed and were indicated with asterisks: *P≤0.05, **P≤0.01 and ***P≤0.001.

## Additional information

**How to cite this article:** Monteiro, J. M. *et al*. Cell shape dynamics during the staphylococcal cell cycle. *Nat. Commun.* 6:8055 doi: 10.1038/ncomms9055 (2015).

## Supplementary Material

Supplementary Information Supplementary Figures 1-6, Supplementary Tables 1-4 and Supplementary References

Supplementary Movie 1 Time lapse of dividing S. aureus cells imaged every 3 minutes S. aureus COL cells were labelled with Nile Red, placed on a medium-containing agarose pad and imaged during 42 minutes, at 3 minutes intervals, by Super-Resolution Structured Illumination Microscopy (SR-SIM). Scale bar 1 μm.

Supplementary Movie 2 Time lapse of dividing S. aureus cells imaged every two milliseconds A 60 ms segment of a 1 minute timelapse of S. aureus COL cells imaged at 2 milliseconds intervals, by Differential Interference Contrast (DIC) microscopy, while growing on a medium-containing agarose pad.

## References

[b1] Tzagoloff H. & Novick R. Geometry of cell division in *Staphylococcus aureus*. J. Bacteriol. 129, 343–350 (1977) . 830642 10.1128/jb.129.1.343-350.1977 PMC234932

[b2] Pinho M. G., Kjos M. & Veening J.-W. How to get (a)round: mechanisms controlling growth and division of coccoid bacteria. Nat. Rev. Microbiol. 11, 601–614 (2013) . 23949602 10.1038/nrmicro3088

[b3] Turner R. D. . Peptidoglycan architecture can specify division planes in *Staphylococcus aureus*. Nat. Commun. 1, 26 (2010) . 20975691 10.1038/ncomms1025

[b4] Scheffers D. J. & Pinho M. G. Bacterial cell wall synthesis: new insights from localization studies. Microbiol. Mol. Biol. Rev. 69, 585–607 (2005) . 16339737 10.1128/MMBR.69.4.585-607.2005 PMC1306805

[b5] Pinho M. & Errington J. Dispersed mode of *Staphylococcus aureus* cell wall synthesis in the absence of the division machinery. Mol. Microbiol. 50, 871–881 (2003) . 14617148 10.1046/j.1365-2958.2003.03719.x

[b6] Seligman S. J. & Pincus M. R. A model for the three-dimensional structure of peptidoglycan in *staphylococci*. J. Theor. Biol. 124, 275–292 (1987) . 3657196 10.1016/s0022-5193(87)80116-9

[b7] Oshida T. . A *Staphylococcus aureus* autolysin that has an N-acetylmuramoyl-L-alanine amidase domain and an endo-beta-N-acetylglucosaminidase domain: cloning, sequence analysis, and characterization. Proc. Natl Acad. Sci. USA 92, 285–289 (1995) . 7816834 10.1073/pnas.92.1.285 PMC42863

[b8] Sugai M. . Identification of endo-beta-N-acetylglucosaminidase and N-acetylmuramyl-L-alanine amidase as cluster-dispersing enzymes in *Staphylococcus aureus*. J. Bacteriol. 177, 1491–1496 (1995) . 7883705 10.1128/jb.177.6.1491-1496.1995 PMC176764

[b9] Yamada S. . An autolysin ring associated with cell separation of *Staphylococcus aureus*. J. Bacteriol. 178, 1565–1571 (1996) . 8626282 10.1128/jb.178.6.1565-1571.1996 PMC177839

[b10] Kuru E. . In situ Probing of Newly Synthesized Peptidoglycan in Live Bacteria with Fluorescent D-Amino Acids. Angew. Chem. Int. Ed. 51, 12519–12523 (2012) . 10.1002/anie.201206749 PMC3589519 23055266

[b11] Amako K., Umeda A. & Murata K. Arrangement of peptidoglycan in the cell wall of *Staphylococcus* spp. J. Bacteriol. 150, 844–850 (1982) . 7068534 10.1128/jb.150.2.844-850.1982 PMC216437

[b12] Amako K. & Umeda A. Scanning electron microscopy of *Staphylococcus*. J. Ultrastruct. Res. 58, 34–40 (1977) . 833916 10.1016/s0022-5320(77)80005-1

[b13] Abbe E. Beitrage zur theorie des mikroskops und der mikroskopischen Wahrnehmung. Arch. Mikrosk. Anat. 9, 413–418 (1873) .

[b14] Rayleigh L. On the theory of optical images, with special reference to microscopy. Philos. Mag. 42, 167–195 (1896) .

[b15] Schermelleh L., Heintzmann R. & Leonhardt H. A guide to super-resolution fluorescence microscopy. J. Cell Biol. 190, 165–175 (2010) . 20643879 10.1083/jcb.201002018 PMC2918923

[b16] Pinho M. G. & Errington J. Recruitment of penicillin-binding protein PBP2 to the division site of *Staphylococcus aureus* is dependent on its transpeptidation substrates. Mol. Microbiol. 55, 799–807 (2005) . 15661005 10.1111/j.1365-2958.2004.04420.x

[b17] Pereira S. F., Henriques A. O., Pinho M. G., de Lencastre H. & Tomasz A. Role of PBP1 in cell division of *Staphylococcus aureus*. J. Bacteriol. 189, 3525–3531 (2007) . 17307860 10.1128/JB.00044-07 PMC1855886

[b18] Pinho M. G., Filipe S. R., de Lencastre H. & al E. Complementation of the essential peptidoglycan transpeptidase function of penicillin-binding protein 2 (PBP2) by the drug resistance protein PBP2A in *Staphylococcus aureus*. J. Bacteriol. 183, 6525–6531 (2001) . 11673420 10.1128/JB.183.22.6525-6531.2001 PMC95481

[b19] Atilano M. . Teichoic acids are temporal and spatial regulators of peptidoglycan cross-linking in *Staphylococcus aureus*. Proc. Natl Acad. Sci. USA 107, 18991–18996 (2010) . 20944066 10.1073/pnas.1004304107 PMC2973906

[b20] Kajimura J. . Identification and molecular characterization of an N-acetylmuramyl-L-alanine amidase Sle1 involved in cell separation of *Staphylococcus aureus*. Mol. Microbiol. 58, 1087–1101 (2005) . 16262792 10.1111/j.1365-2958.2005.04881.x

[b21] Ramadurai L., Lockwood K. J., Nadakavukaren M. J. & Jayaswal R. K. Characterization of a chromosomally encoded glycylglycine endopeptidase of *Staphylococcus aureus*. Microbiology 145, 801–808 (1999) . 10220159 10.1099/13500872-145-4-801

[b22] Dubrac S., Boneca I. G., Poupel O. & Msadek T. New insights into the WalK/WalR (YycG/YycF) essential signal transduction pathway reveal a major role in controlling cell wall metabolism and biofilm formation in *Staphylococcus aureus*. J. Bacteriol. 189, 8257–8269 (2007) . 17827301 10.1128/JB.00645-07 PMC2168699

[b23] Pereira P., Veiga H., Jorge A. & Pinho M. fluorescent reporters for studies of cellular localization of proteins in *Staphylococcus aureus*. Appl. Environ. Microbiol. 76, 4346–4353 (2010) . 20453129 10.1128/AEM.00359-10 PMC2897443

[b24] Giesbrecht P., Kersten T., Maidhof H. & Wecke J. Staphylococcal cell wall: morphogenesis and fatal variations in the presence of penicillin. Microbiol. Mol. Biol. Rev. 62, 1371–1414 (1998) . 9841676 10.1128/mmbr.62.4.1371-1414.1998 PMC98950

[b25] Touhami A., Jericho M. H. & Beveridge T. J. Atomic force microscopy of cell growth and division in *Staphylococcus aureus*. J. Bacteriol. 186, 3286–3295 (2004) . 15150213 10.1128/JB.186.11.3286-3295.2004 PMC415778

[b26] Gautam S., Kim T. & Spiegel D. A. Chemical probes reveal an extraseptal mode of cross-linking in *Staphylococcus aureus*. J. Am. Chem. Soc. 137, 7441–7447 (2015) . 26035224 10.1021/jacs.5b02972

[b27] Qiao Y. . Detection of lipid-linked peptidoglycan precursors by exploiting an unexpected transpeptidase reaction. J. Am. Chem. Soc. 136, 14678–14681 (2014) . 25291014 10.1021/ja508147s PMC4210121

[b28] Memmi G., Filipe S., Pinho M., Fu Z. & Cheung A. *Staphylococcus aureus* PBP4 Is essential for beta-lactam resistance in community-acquired methicillin-resistant strains. Antimicrob. Agents Chemother. 52, 3955–3966 (2008) . 18725435 10.1128/AAC.00049-08 PMC2573147

[b29] Leski T. A. & Tomasz A. Role of penicillin-binding protein 2 (PBP2) in the antibiotic susceptibility and cell wall cross-linking of *Staphylococcus aureus*: evidence for the cooperative functioning of PBP2, PBP4, and PBP2A. J. Bacteriol. 187, 1815–1824 (2005) . 15716453 10.1128/JB.187.5.1815-1824.2005 PMC1064008

[b30] Loskill P. . Reduction of the peptidoglycan crosslinking causes a decrease in stiffness of the *Staphylococcus aureus* cell envelope. Biophys. J. 107, 1082–1089 (2014) . 25185544 10.1016/j.bpj.2014.07.029 PMC4156677

[b31] Matias V. R. & Beveridge T. J. Native cell wall organization shown by cryo-electron microscopy confirms the existence of a periplasmic space in *Staphylococcus aureus*. J. Bacteriol. 188, 1011–1021 (2006) . 16428405 10.1128/JB.188.3.1011-1021.2006 PMC1347357

[b32] Zhou X. . Bacterial division. Mechanical crack propagation drives millisecond daughter cell separation in *Staphylococcus aureus*. Science 348, 574–578 (2015) . 25931560 10.1126/science.aaa1511 PMC4864021

[b33] Bailey R. G. . The interplay between cell wall mechanical properties and the cell cycle in *Staphylococcus aureus*. Biophys. J. 107, 2538–2545 (2014) . 25468333 10.1016/j.bpj.2014.10.036 PMC4255174

[b34] Veiga H., Jorge A. M. & Pinho M. G. Absence of nucleoid occlusion effector Noc impairs formation of orthogonal FtsZ rings during *Staphylococcus aureus* cell division. Mol. Microbiol. 80, 1366–1380 (2011) . 21477126 10.1111/j.1365-2958.2011.07651.x

[b35] Arnaud M., Chastanet A. & Debarbouille M. New vector for efficient allelic replacement in naturally nontransformable, low-GC-content, gram-positive bacteria. Appl. Environ. Microbiol. 70, 6887–6891 (2004) . 15528558 10.1128/AEM.70.11.6887-6891.2004 PMC525206

[b36] Atilano M. L. . Bacterial autolysins trim cell surface peptidoglycan to prevent detection by the Drosophila innate immune system. eLife 3, e02277 (2014) . 24692449 10.7554/eLife.02277 PMC3971415

[b37] Christie G. E. . The complete genomes of *Staphylococcus aureus* bacteriophages 80 and 80alpha--implications for the specificity of SaPI mobilization. Virology 407, 381–390 (2010) . 20869739 10.1016/j.virol.2010.08.036 PMC2952651

[b38] Wu S., de Lencastre H., Sali A. & Tomasz A. A phosphoglucomutase-like gene essential for the optimal expression of methicillin resistance in *Staphylococcus aureus*: molecular cloning and DNA sequencing. Microb. Drug Resist. 2, 277–286 (1996) . 9158773 10.1089/mdr.1996.2.277

[b39] Henner D. J. Inducible expression of regulatory genes in *Bacillus subtilis*. Methods Enzymol. 185, 223–228 (1990) . 2116574 10.1016/0076-6879(90)85022-g

[b40] Vagner V., Dervyn E. & Ehrlich S. D. A vector for systematic gene inactivation in *Bacillus subtilis*. Microbiology 144, 3097–3104 (1998) . 9846745 10.1099/00221287-144-11-3097

[b41] Subach F. V. . Photoactivatable mCherry for high-resolution two-color fluorescence microscopy. Nat. Methods 6, 153–159 (2009) . 19169259 10.1038/nmeth.1298 PMC2901231

[b42] Charpentier E. . Novel cassette-based shuttle vector system for gram-positive bacteria. Appl. Environ. Microbiol. 70, 6076–6085 (2004) . 15466553 10.1128/AEM.70.10.6076-6085.2004 PMC522135

[b43] Gill S. R. . Insights on evolution of virulence and resistance from the complete genome analysis of an early methicillin-resistant *Staphylococcus aureus* strain and a biofilm-producing methicillin-resistant *Staphylococcus epidermidis* strain. J. Bacteriol. 187, 2426–2438 (2005) . 15774886 10.1128/JB.187.7.2426-2438.2005 PMC1065214

[b44] Heintzmann R. & Cremer C. G. Laterally modulated excitation microscopy: improvement of resolution by using a diffraction gratingin Proceedings of the SPIE3568 (Optical Biopsies and Microscopic Techniques III) 185–196Stockholm, Sweden (1999) .

[b45] Kuru E., Tekkam S., Hall E., Brun Y. V. & Van Nieuwenhze M. S. Synthesis of fluorescent D-amino acids and their use for probing peptidoglycan synthesis and bacterial growth in situ. Nat. Protoc. 10, 33–52 (2015) . 25474031 10.1038/nprot.2014.197 PMC4300143

[b46] Xu D. . The ellipsoidal area ratio: an alternative anisotropy index for diffusion tensor imaging. Magn. Reson. Imaging 27, 311–323 (2009) . 18835122 10.1016/j.mri.2008.07.018 PMC3575168

[b47] Pereira P., Filipe S., Tomasz A. & Pinho M. Fluorescence ratio imaging microscopy shows decreased access of vancomycin to cell wall synthetic sites in vancomycin-resistant staphylococcus aureus. Antimicrob. Agents Chemother. 51, 3627–3633 (2007) . 17646417 10.1128/AAC.00431-07 PMC2043281

