# Supplementary material for: Cell shape dynamics during the staphylococcal cell cycle
Source: Nat Commun. 2015 Aug 17;6:8055. doi: 10.1038/ncomms9055 (PMC4557339; doi:10.1038/ncomms9055)
Supplement: Supplementary Information — Supplementary Figures 1-6, Supplementary Tables 1-4 and Supplementary References [file ncomms9055-s1.pdf]

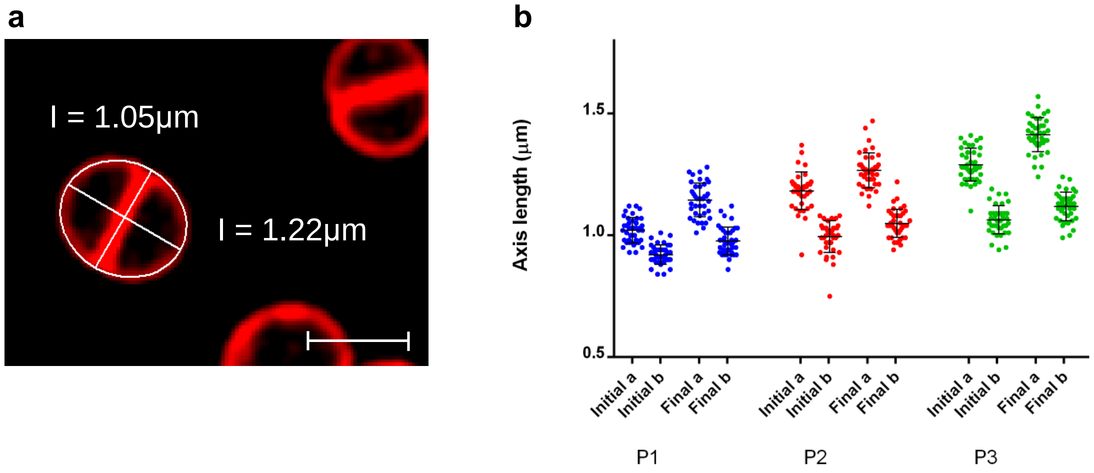

Supplementary Figure 1: Size measurements of *S. aureus* cells imaged by SR-SIM during the cell cycle

(a) The shape of *S. aureus* cells was approximated to a prolate spheroid and superimposition of an elipse was used to measure the longer and shorter cellular axes during the different phases of growth. Scale bar = 1 µm

(b) Scatter plot of the measurements of the longer (a) and shorter (b) axes of *S. aureus* cells when starting (Initial) and finishing (Final) each of the three growth phases defined in Figure 1. Black lines represent mean±s.d.

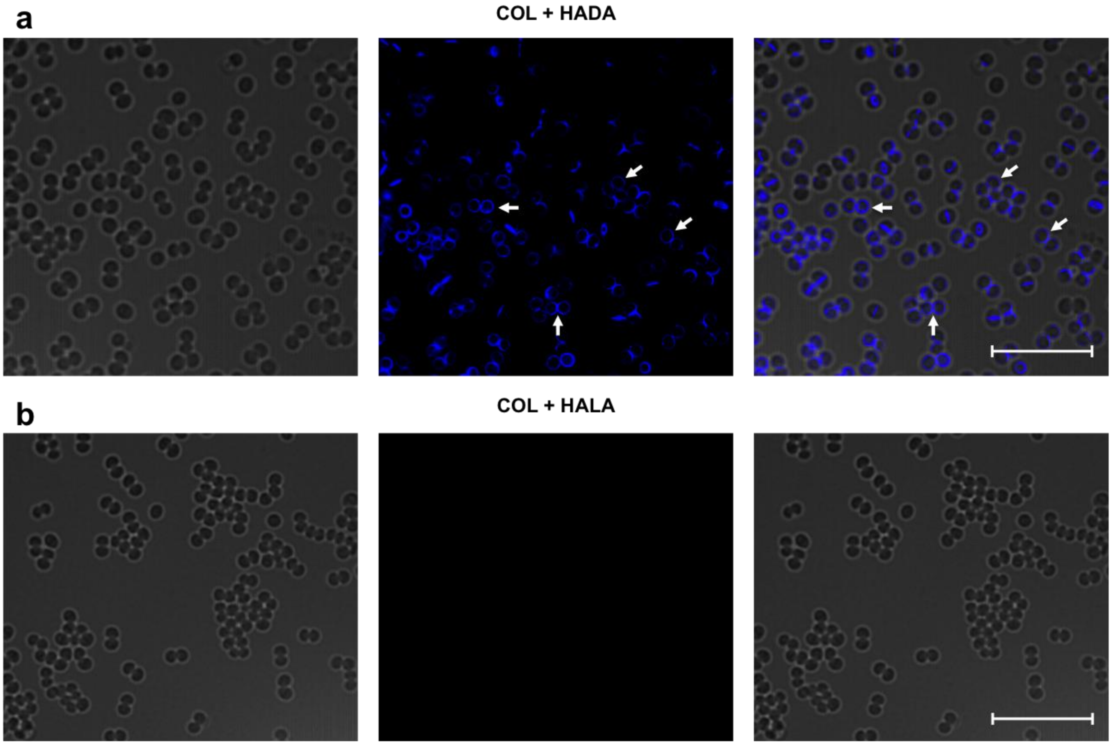

Supplementary Figure 2: Fluorescent D-amino acid HADA is incorporated at the peripheral wall of *S. aureus* cells

S. aureus COL cells were labelled for 5 minutes with HADA, a fluorescent derivative of 3-amino-D-alanine that can be incorporated in the pentapeptide chain of peptidoglycan (a) or its L-enantiomer HALA that should not be incorporated into the peptidoglycan (b), used as a control for non-specific binding. Cells were then imaged by SR-SIM, showing that the peripheral walls of cells in phase 1 (examples indicated by white arrows) were labelled by HADA but not by HALA. Scale bars = 10 μm

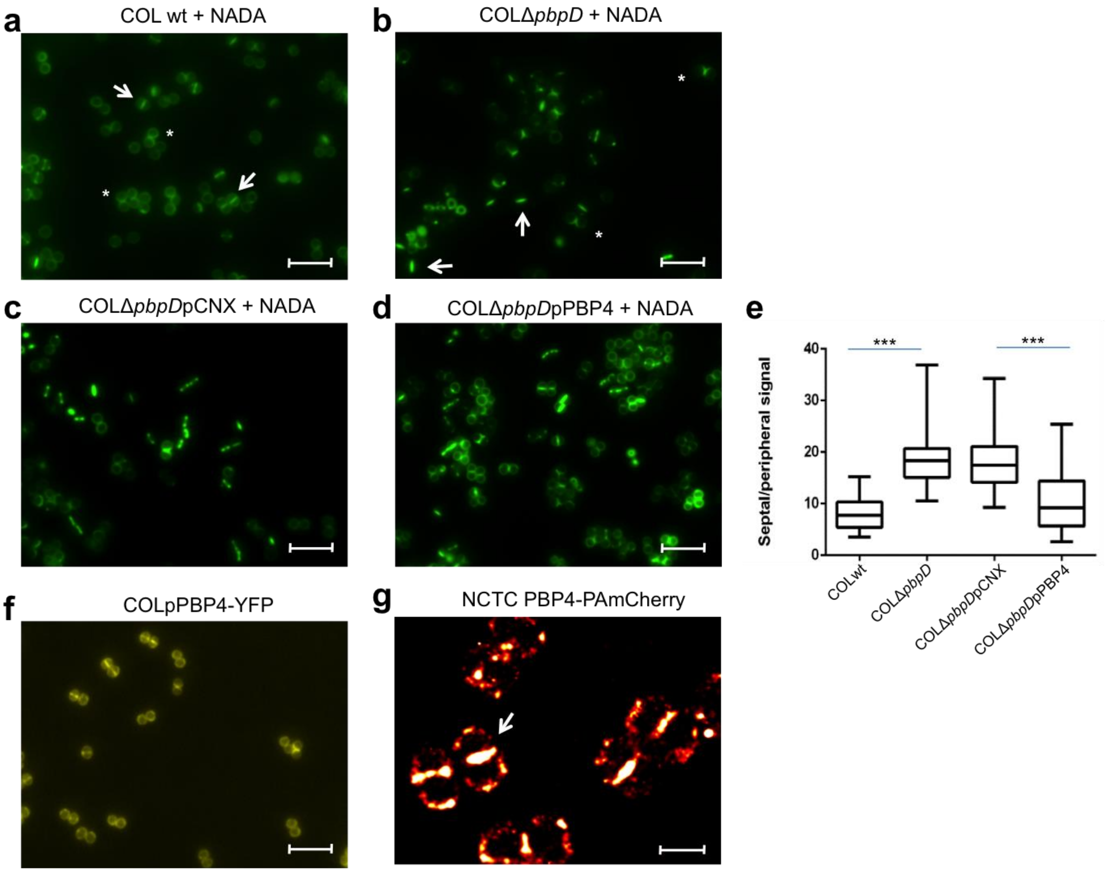

Supplementary Figure 3: PBP4 is active at the division septum and at the peripheral wall.

(a) *S. aureus* COL cells were incubated for 20 minutes with NADA and imaged by wide-field fluorescence microscopy. Cells with partial (Phase 2) or complete (Phase 3) septa (arrows) showed labelling mostly at the septum, although some fluorescent signal could also be observed at the peripheral cell surface**.** Cells without septa (Phase 1, asterisks) showed NADA incorporation at the peripheral cell wall. Scale bar = 5 µm.

(b) COLΔ*pbpD* cells, lacking PBP4 were imaged as described above. NADA incorporation at the peripheral cell surface was virtually absent both in cells undergoing septation (arrows) and in newly split cells (asterisk), indicating that PBP4 is responsible for the majority of peripheral NADA incorporation. Scale bar = 5 µm.

(c,d) Strain COLΔ*pbpD* was complemented with empty pCNX vector (c) and pCNX encoding PBP4 under the control of P_{Cad} (d), in the presence of cadmium chloride (1µM), and imaged as above. NADA incorporation at the peripheral cell surface was only observed in the strain complemented with PBP4. Scale bar = 5 µm.

(**e**) The intensity of the NADA fluorescence signal was measured at the septum and peripheral wall, in cells (n=50) with complete division septa, and the ratio between septal and peripheral signal was calculated for each cell. This ratio was significantly higher for COLΔ*pbpD* than for COL cells (18.7±5.4 vs 7.9±3.0, $p<0.001$) showing that the mutant lacking PBP4 is deficient in peripheral NADA incorporation. This ratio was also higher in COLΔ*pbpD* containing the empty vector pCNX than in the same strain complemented with plasmid-encoded PBP4 (18.2±5.7 vs 10.2±5.4, $p<0.001$). Data is represented as box-and-whisker plots where boxes correspond to the first to third quartiles, lines inside the boxes indicate the median and ends of whiskers represent the

minimum and maximum of all data. Statistical analysis was performed using the unpaired *t* test (***p<0.001).

(**f**) Localization of a YFP-tagged derivative of PBP4 in *S. aureus* COL cells by wide-field fluorescence microscopy showing that a small fraction of PBP4 localizes at the cell periphery. Scale bar = 5 µm.

(g) PALM image showing PBP4-PAmCherry localization in *S. aureus* NCTC8325-4 background. Cells (n=50) showing PBP4-PAmCherry in complete septa (white arrow) were used to quantify the number of PBP4 molecules (n_{total}=27533) present either at the peripheral membrane (26±11%) or at the septa (74±11%). Images are false colored showing red to white increasing intensity range. Scale bar 1µm.

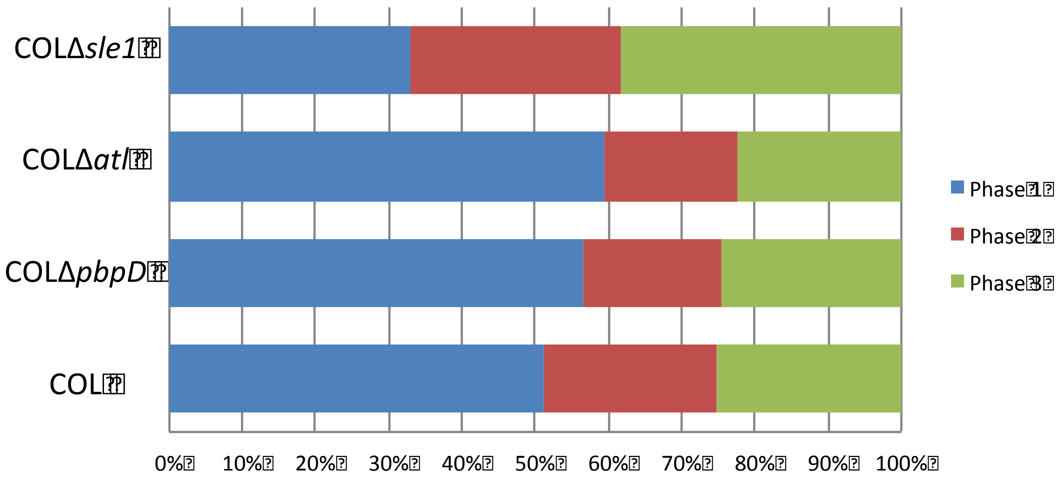

Supplementary Figure 4: Effect of impaired peptidoglycan synthesis or autolysis on the cell cycle of *S. aureus.*

Aliquots of exponentially liquid growing cultures, at 37ºC, of the parental strain COL (n=427, T_d= 36 minutes) and mutants lacking peptidoglycan synthesis enzyme PBP4 (COLΔ*pbpD,* n=304, T_d= 36 minutes) or autolysins Atl (COLΔ*atl,* n=461, T_d= 40 minutes)) and Sle1 (COLΔ*sle1,* n=588, T_d= 38 minutes) were placed on agarose pads and immediately imaged by SR-SIM. The percentage of cells in each phase of the cell cycle is shown in the graph and corresponds to the fraction of the cell cycle spent in each phase. T_d, duplication time calculated during growth in liquid culture.

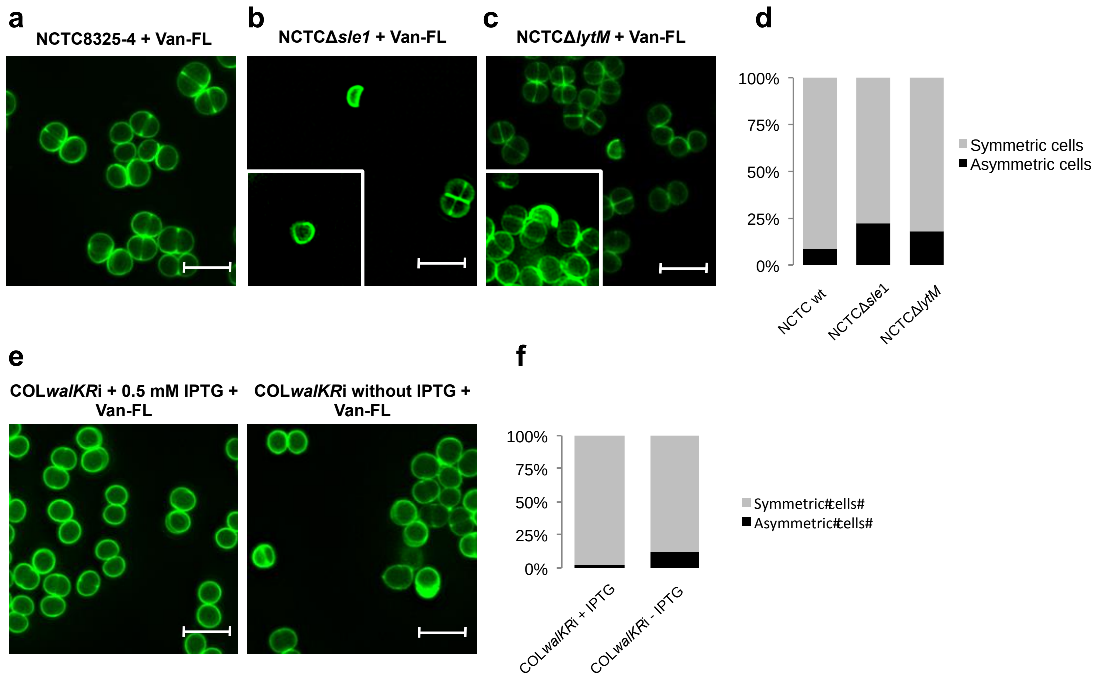

Supplementary Figure 5: Effect of impaired peptidoglycan autolysis on the morphology of *S. aureus.*

Cells of MSSA strain NCTC8325-4 **(a)**, its mutant lacking autolysin Sle1 (NCTCΔ*sle1*, **(b)**), or lacking autolysin LytM (NCTCΔ*lytM,* **(c)**) were labelled with cell wall dye Van-FL and imaged by SR-SIM, confirming the presence of D-shaped cells in the mutants.

(d) Symmetry of cells depicted in panels "a" to "c" was assessed as described in Fig 6d, showing an increase in the frequency of asymmetric cells in the two autolysin mutants.

(e) Cells of MRSA strain COL with the two-component system *walKR* (positive regulator of autolysins) placed under the control of the IPTG-inducible promoter P*spac* (COL*walKR*i), were grown in the presence or in the absence of inducer, labelled with Van-FL and imaged by SR-SIM, showing the presence of D-shaped cells.

(f) Symmetry of cells depicted in panel "e" was assessed as described in Fig 6d, showing an increase in the frequency of asymmetric cells upon depletion of the WalKR two-component system.

Scale bars = 2 μm

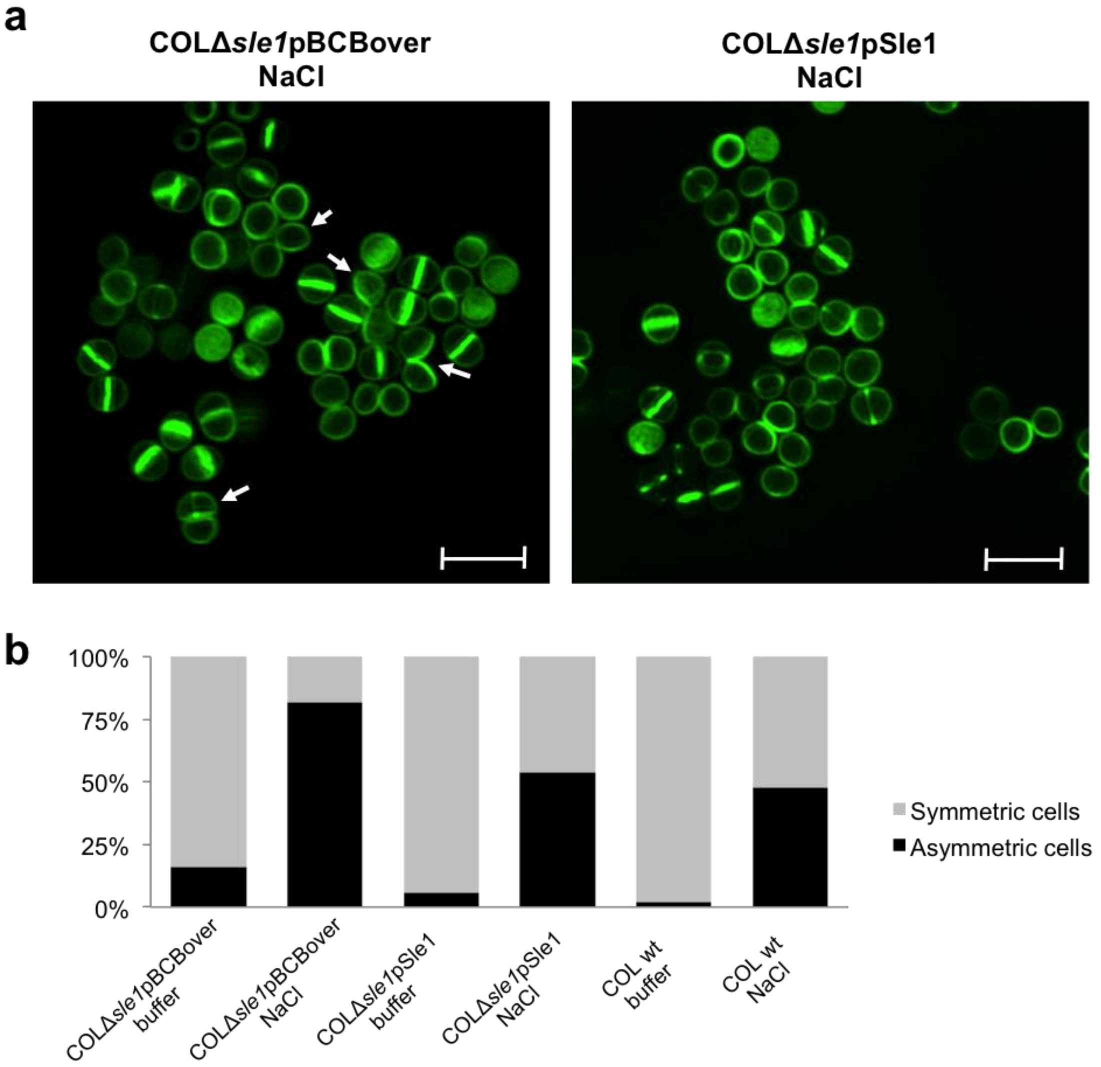

Supplementary Figure 6: Complementation of COLΔ*sle1* with plasmid-encoded Sle1

(a) Cells of COLΔ*sle1* strain were transformed with empty vector pBCBover (left) or the same vector encoding *sle1* under the control of IPTG inducible P_{spac} promoter (right) were labelled with NADA, incubated for 15 minutes in saturating concentration of NaCl, placed on an agarose pad containing the same salt concentration and imaged by SR-SIM. Asymmetric cells with a shape close to a "D" (white arrows) were observed in higher frequency in the strain containing the empty vector. Scale bars = 2 μm.
(b) Symmetry of cells depicted in panel "a" was assessed as described in Fig 6d, showing that the shape phenotype of strain COLΔ*sle1* is complemented by the expression of Sle1 from a plasmid.

Supplementary Table 1: Average ratio of longer/shorter axes of *S. aureus* cells upon initiation and completion of the three phases of the cell cycle.

Strain	P1		P2		P3	
	Initial	Final	Initial	Final	Initial	Final
COL	1.11±0.04	1.17±0.06	1.19±0.05	1.21±0.04	1.22±0.06	1.27±0.05
COLΔ*pbpD*	1.13±0.09	1.17±0.07	1.20±0.06	1.23±0.05	1.22±0.04	1.28±0.06
COLΔ*atl*	1.13±0.07	1.14±0.05 (**)	1.15±0.06 (**)	1.17±0.06 (***)	1.17±0.06 (**)	1.23±0.05 (*)
COLΔ*sle1*	1.19±0.08 (***)	1.20±0.08	1.19±0.06	1.19±0.06	1.20±0.07	1.23±0.10

* $p \leq 0.05$, ** $p \leq 0.01$, *** $p \leq 0.001$ mutants vs. parental strain COL (n=40 cells for each strain). Data are presented as mean±s.d. Statistical analysis was performed using the unpaired *t* test.

Supplementary Table 2: Average volume (µm^3) of *S. aureus* cells upon initiation and completion of the three phases of the cell cycle.

Strain	P1		P2		P3	
	Initial	Final	Initial	Final	Initial	Final
COL	0.47±0.07	0.62±0.08	0.63±0.10	0.72±0.10	0.73±0.10	0.91±0.12
COLΔ*pbpD*	0.40±0.08 (***)	0.59±0.10	0.61±0.08	0.69±0.09	0.70±0.07	0.86±0.11
COLΔ*atl*	0.46±0.07	0.66±0.10	0.68±0.10 (*)	0.79±0.10 (**)	0.82±0.13 (***)	0.97±0.16
COLΔ*sle1*	0.48±0.07	0.55±0.06 (***)	0.55±0.07 (***)	0.64±0.09 (***)	0.63±0.09 (***)	0.98±0.17 (*)

* $p \leq 0.05$, ** $p \leq 0.01$, *** $p \leq 0.001$ mutants vs. parental strain COL (n=40 cells for each strain). Data are presented as mean±s.d. Statistical analysis was performed using the unpaired *t* test .

Supplementary Table 3: Bacterial strains and plasmids used in this study

Strains	Description	Source or reference
Escherichia coli		
DC10B	Δ*dcm* in the DH10B background; Dam methylation only	[1]
Staphylococcus aureus		
COL	HA-MRSA	[2]
NCTC8325-4	MSSA strain	R. Novick
RN4220	Restriction-deficient derivative of NCTC8325-4	R. Novick
COLΔ*pbpD*	*pbp4* in-frame deletion mutant of parental strain COL	[3]
COLpPBP4-YFP	COL encoding a C-terminal YFP fusion to PBP4 at the native locus	[4]
COLΔ*atl*	Δ*atl* in-frame deletion mutant of parental strain COL	This study
COLΔ*sle1*	Δ*sle1* in-frame deletion mutant of parental strain COL	This study
COLΔ*sle1*pBCBover	COLΔ*sle1* with pBCBover	This study
COLΔ*sle1*pSle1	COLΔ*sle1* complemented with pSle1	This study
COL*walKR*i	COL strain with WalKR operon under the control of the IPTG inducible Pspac promoter; Ery^r	This study
NCTCΔ*lytM*	Δ*lytM* in-frame deletion mutant of parental strain NCTC8325-4	This study
NCTCΔ*sle1*	Δ*sle1* in-frame deletion mutant of parental strain NCTC8325-4	This study
NCTCΔ*pbpD::pbpD-PAmCherry*	NCTC strain with the *pbpD* gene substituted by the *pbpD-PAmCherry* photoactivable derivative	This study
BCBPM120	COLΔ*pbpD* with pCNX	This study
BCBPM138	COLΔ*pbpD* with pBCBPM115, encoding PBP4 under the control of P_{Cad}	This study
Sporosarcina ureae SL6708	Derivative of *S. ureae* ATCC 1388	[5]

Supplementary Table 3 (cont): Bacterial strains and plasmids used in this study

**Plasmids**		
pMAD	*E. coli-S. aureus* shuttle vector with a thermosensitive origin of replication for Gram-positive bacteria; $Amp^r Ery^r$ *lacZ*	[6]
pMUTIN4	Integrative vector for *S.aureus* encoding IPTG inducible P_{spac} promoter; Amp^r, Ery^r	[7]
pCN34	Vector containing the aphA-3 kanamycin resistance cassette	[8]
pCN51	Shuttle vector containing a cadmium inducible P_{cad} promoter; Amp^R Ery^R	[8]
pCNX	Shuttle vector containing a cadmium inducible P_{cad} promoter; Amp^R Kan^R	This study
pGC2	*E. coli/S. aureus* shuttle vector, Amp^r Cm^r	[9]
pDH88	*B. subtilis* with P_{spac} promoter and *lacI*	[10]
pBAD/HisB-PAmCherry1	Plasmid encoding the sequence of the photoactivable PAmCherry1 protein	[11]
pΔ*atl*	pMAD derivative with the up- and downstream regions of *atl*	[12]
pΔ*lytM*	pMAD derivative with the up- and downstream regions of *lytM*	This study
pΔ*sle1*	pMAD derivative with the up- and downstream regions of *sle1*	This study
pBCBover	pGC2 derivative containg P_{spac} promoter and *lacI*	This study
pSle1	pBCBover encoding Sle1 under the control of P_{spac} promoter	This study
p*walKR*	pMUTIN4 derivative containing the ribosome binding site and 5' end of *walKR*	This study
pBCBRP007	pCNX derivative containing P_{cad}-RBS-*pbpD*-7aaLinker-*PAmCherry*; Amp^R Kan^R	This study
pBCBRP008	pMAD derivative containing truncated *pbpD*-7aa-*PAmCherry*-Downstream region; Amp^R Ery^R	This study
pBCBPM115	pCNX encoding PBP4 under the control of P_{cad}	This study

Supplementary Table 4: Primers used in this study

Primer name	Sequence (5'-3')
P1_Sle1	CATGCCATGGGCAGTAGATGCACAACAAACTG
P2_Sle1	CATTATATATTTATATACGTAAGACTTTATTTAAAATCCTCCTCTTGCTTAAC
P3_Sle1	GTTAAGCAAGAGGAGGATTTTAAATAAAGTCTTACGTATATAAATATATAATG
P4_Sle1	TGGAGATCTCAGCGCGTGTACTTGTGATTC
P1_lytM	CATGCCATGGGCAATGAAGCAGGTACATTTG
P2_lytM	GCAACTTGGGATTTTCTGTATTAGTATAAAACATCCTCCATTAAAG
P3_lytM	CTTTAATGGAGGATGTTTTATACTAATACAGAAAATCCCAAGTTGC
P4_lytM	TGGAGATCT GGAGCGTAACTGATGATAG
yycF fw EcoRI	GCGCGCGAATTCTATTAATGATTTAAGAAAAGAGG
yycF stop rv BamHI	CGCGCGCGGATCCCTAGCCACGTTTTTTAATAGAATATGCG
P1pbp4pCNX	GGATCCAGGAGGTACCTTATGAAAAATTTAATATC
P2pbp4pCNX	CACCATGCTAGCGGCGCGCCGGGTACCTTTTCTTTTTCTAAATAAACGATTGATTA
P3pbp4pCNX	AGGTACCCGGCGCGCCGCTAGCATGGTGAGCAAGGGCGAGGA
P4pbp4pCNX	GCCTAAGAATTCTTACTTGTACAGCTCGTCCATGC
P1pMADpbp4PAmCherry	GATATCGGATCCACAGTCACAATGACGAACAAAG
P2pMADpbp4PAmCherry	GTCCGTTTTTAGTATGTTTTACTTGTACAGCTCGTCCATGCCG
P3pMADpbp4PAmCherry	TGGACGAGCTGTACAAGTAAAACATACTAAAAACGGACAAGTTGC
P4pMADpbp4PAmCherry	ATGGTACCCGGGACAAGTAACGATGAAGATTTTAATAG
Pspac_pDH88_P9_XhoI_SalI	GCTGCGCTGTCGACGTTCACCTCGAGTTCTACACAGCCCAGTCCAGAC
Pspac_pDH88_SpeI_P10	GTTAACAAAGACTAGTATGCTCTAGAAACCCGGGAAAAGC
Pspac_pDH88_SpeI_P11	CTAGAGCATACTAGTCTTTGTTAACTTAGATCTTTATCG
Pspac_pDH88_P12_XhoI_EcoRI	GCTGAATTCGATGCCCTCGAGCTGATCCTAACTCACATTAATTGCG
Sle1_FW_XmaI	TACTCCCCGGGGCAAGAGGAGGATTTTAAAGTGC
Sle1_RV_XbaI	GCTGCTCTAGATTAGTGAATATATCTATAATTATTTAC
PBP4pCadP1BamFW	CGCAGGATCCAGGAGGTACCTTATGAAAAATTTAATATCTATTATC
pCADPBP4wt_EcoREV	GCGTGAATTCTTAGGTACCTTATTTTCTTTTTCTAAATAAAC

Underlined sequences correspond to restriction sites

SUPPLEMENTARY REFERENCES

1 Monk, I. R., Shah, I. M., Xu, M., Tan, M. W. & Foster, T. J. Transforming the untransformable: application of direct transformation to manipulate genetically *Staphylococcus aureus* and *Staphylococcus epidermidis*. *mBio* **3**, doi:10.1128/mBio.00277-11 (2012).

2 Gill, S. R. *et al.* Insights on evolution of virulence and resistance from the complete genome analysis of an early methicillin-resistant *Staphylococcus aureus* strain and a biofilm-producing methicillin-resistant *Staphylococcus epidermidis* strain. *J. Bacteriol.* **187**, 2426-2438 (2005).

3 Memmi, G., Filipe, S., Pinho, M., Fu, Z. & Cheung, A. *Staphylococcus aureus* PBP4 Is Essential for beta-Lactam Resistance in Community-Acquired Methicillin-Resistant Strains. *Antimicrob. Agents. Chemother.* **52**, 3955-3966 (2008).

4 Loskill, P. *et al.* Reduction of the peptidoglycan crosslinking causes a decrease in stiffness of the *Staphylococcus aureus* cell envelope. *Biophys. J.* **107**, 1082-1089 (2014).

5 Zhang, L., Higgins, M. L. & Piggot, P. J. The division during bacterial sporulation is symmetrically located in *Sporosarcina ureae*. *Mol. Microbiol.* **25**, 1091-1098 (1997).

6 Arnaud, M., Chastanet, A. & Debarbouille, M. New vector for efficient allelic replacement in naturally nontransformable, low-GC-content, gram-positive bacteria. *Appl. Environ. Microbiol.* **70**, 6887-6891, (2004).

7 Vagner, V., Dervyn, E. & Ehrlich, S. D. A vector for systematic gene inactivation in *Bacillus subtilis*. *Microbiology* **144**, 3097-3104 (1998).

8 Charpentier, E. *et al.* Novel cassette-based shuttle vector system for gram-positive bacteria. *Appl. Environ. Microbiol.* **70**, 6076-6085 (2004).

9 Wu, S., de Lencastre, H., Sali, A. & Tomasz, A. A phosphoglucomutase-like gene essential for the optimal expression of methicillin resistance in *Staphylococcus aureus*: molecular cloning and DNA sequencing. *Microb Drug Resist* **2**, 277-286 (1996).

10 Henner, D. J. Inducible expression of regulatory genes in *Bacillus subtilis*. *Methods Enzymol* **185**, 223-228 (1990).

11 Subach, F. V. *et al.* Photoactivatable mCherry for high-resolution two-color fluorescence microscopy. *Nature methods* **6**, 153-159 (2009).

12 Atilano, M. L. *et al.* Bacterial autolysins trim cell surface peptidoglycan to prevent detection by the Drosophila innate immune system. *eLife* **3**, e02277, doi:10.7554/eLife.02277 (2014).
